# Alfalfa snakin-1 prevents fungal colonization and probably coevolved with rhizobia

**DOI:** 10.1186/s12870-014-0248-9

**Published:** 2014-09-17

**Authors:** Araceli Nora García, Nicolás Daniel Ayub, Ana Romina Fox, María Cristina Gómez, María José Diéguez, Elba María Pagano, Carolina Andrea Berini, Jorge Prometeo Muschietti, Gabriela Soto

**Affiliations:** Instituto de Genética Ewald A. Favret (CICVyA-INTA), De los Reseros S/N, Castelar, C25 (1712) Buenos Aires Argentina; Instituto de Investigaciones Biomédicas en Retrovirus y SIDA (INBIRS), UBA-CONICET, Paraguay 2155, C1121ABG Ciudad Autónoma de Buenos Aires, Argentina; Departamento de Biodiversidad y Biología Experimental, Facultad de Ciencias Exactas y Naturales, Universidad de Buenos Aires, Intendente Güiraldes 2160, Ciudad Universitaria, Pabellón II, C1428EGA Ciudad Autónoma de Buenos Aires, Argentina; Instituto de Investigaciones en Ingeniería Genética y Biología Molecular, “Dr. Hector Torres”, (INGEBI-CONICET), Vuelta de Obligado 2490, C1428ADN Ciudad Autónoma de Buenos Aires, Argentina

**Keywords:** Antimicrobial peptides, Alfalfa, Evolution, Land plants, Innate immunity, Snakin

## Abstract

**Background:**

The production of antimicrobial peptides is a common defense strategy of living cells against a wide range of pathogens. Plant snakin peptides inhibit bacterial and fungal growth at extremely low concentrations. However, little is known of their molecular and ecological characteristics, including origin, evolutionary equivalence, specific functions and activity against beneficial microbes. The aim of this study was to identify and characterize snakin-1 from alfalfa (*Ms*SN1).

**Results:**

Phylogenetic analysis showed complete congruence between snakin-1 and plant trees. The antimicrobial activity of *Ms*SN1 against bacterial and fungal pathogens of alfalfa was demonstrated *in vitro* and *in vivo.* Transgenic alfalfa overexpressing *MsSN1* showed increased antimicrobial activity against virulent fungal strains*.* However, *Ms*SN1 did not affect nitrogen-fixing bacterial strains only when these had an alfalfa origin.

**Conclusions:**

The results reported here suggest that snakin peptides have important and ancestral roles in land plant innate immunity. Our data indicate a coevolutionary process, in which alfalfa exerts a selection pressure for resistance to *Ms*SN1 on rhizobial bacteria. The increased antimicrobial activity against virulent fungal strains without altering the nitrogen-fixing symbiosis observed in *MsSN1*-overexpressing alfalfa transgenic plants opens the way to the production of effective legume transgenic cultivars for biotic stress resistance.

**Electronic supplementary material:**

The online version of this article (doi:10.1186/s12870-014-0248-9) contains supplementary material, which is available to authorized users.

## Background

Alfalfa (*Medicago sativa* L.), known as the “Queen of Forages”, is a perennial legume. This species is native to Asia, and is considered one of the first known crops with a cultivation history of at least 3500 years. Due to its strong vitality, high nutritional quality, high yields, high adaptability and multiple uses, alfalfa is the main forage crop produced in temperate regions of the planet. Elite alfalfa cultivars must not only have high forage yields but also maintain their productivity and stands over several years to provide substantial economic benefits. Regarding this complex topic, improved fungal disease resistance has been specifically identified as the critical trait in alfalfa persistence [1]. In this context, it is proposed that the use of snakin-1 peptide (SN1), a powerful but poorly studied antimicrobial compound, to improve alfalfa tolerance to virulent fungal pathogens should be explored.

Antimicrobial peptides are present in virtually all organisms and are an ancient and critical component of innate immunity. SN1, the first member of the snakin family to be characterized, was isolated from a crude cell wall preparation of potato (*Solanum tuberosum*) tubers (*St*SN1) [2]. This cysteine-rich peptide from potato was found to be active against bacterial and fungal pathogens at extremely low concentrations (EC_50_ < 10 μM) [3]. The expression pattern of the *StSN1* gene suggests that plant SN1 could be a component of constitutive defense barriers, especially those of storage and reproductive plant organs [3]. A second snakin peptide (*St*SN2), which has a high amino acid identity (30%) to *St*SN1, was also isolated from a crude cell wall preparation of potato tubers. Consistent with this high amino acid identity, *St*SN2 is also active at very low concentrations against a wide range of pathogens [4]. In contrast to *StSN1*, the expression of *StSN2* is locally induced by wounding and pathogen infection, suggesting a critical role of snakin-2 in both constitutive and inducible defense barriers of plants. These strong antimicrobial activities of snakin peptides have been verified using bacterial and eukaryotic heterologous expression systems [5-7]. Based on the presence of the Gibberellic Acid Stimulated Arabidopsis (GASA) domain and the absence of bioinformatic (e.g. RGD residues) and functional (e.g. toxic activity) data supporting its relationship with cysteine-rich peptides from snakes venoms, *St*SN1 and *St*SN2 have been recently renamed as GSL1 and GSL2, respectively [8,9].

Overexpression of SN1 in potato and wheat (*Triticum aestivum*), SN2 in potato and tomato (*Solanum lycopersicum*), and snakin-defensin hybrid protein in tobacco (*Nicotiana tabacum*) and potato restricts pathogen invasiveness and enhances tolerance to bacterial and fungal diseases, without altering the agronomic phenotype of these crops [6,9-12]. Furthermore, disease sensitivity is enhanced by silencing SN2 in wild tobacco (*Nicotiana benthamiana*), supporting the central role of snakin peptides in plant defense [13]. In addition, it has been recently shown that *St*SN1 is located in the plant cell wall [14], confirming that snakin peptides are components of the physical barrier and the first line of defense used by plant cells to prevent bacterial and fungal entry [3]. Furthermore, in concordance with the functional classification of snakin peptides as members of the GASA protein family [4,15,16], *StSN1* silencing affects cell division, primary metabolism, and cell wall composition [14]. Snakin peptides could have additional functions in plant growth and development beyond their demonstrated function in biotic stress response. In spite of their hypothetical functional similarity, there is little to no phylogenetic reports on the relationship between *St*SN1 and GASA-related proteins.

The aim of this study was to identify and characterize snakin-1 (*MsSN1*) gene of alfalfa. The phylogenetic and functional analyses showed here, propose that *MsSN1* is an ancestral plant gene involved in biotic stress resistance, suggesting a coevolutionary process, in which alfalfa exerts a selection pressure for resistance to *Ms*SN1 on rhizobial bacteria.

## Methods

### Bacterial and fungal strains

The bacterial strains (all Gram-negative bacteria) used in this study were: *Pseudomonas fluorescens* Pf-5 [17], *Sinorhizobium meliloti* BL225C [18], *Sinorhizobium meliloti* SM11 [19], *Sinorhizobium medicae* WSM419 [20], *Sinorhizobium fredii* USDA 257 [21], *Rhizobium* sp. Or 191 [22], *Rhizobium etli* CFN 42 [23], *Mesorhizobium loti* MAFF303099 [24], *Bradyrhizobium japonicum* USDA110 [25] and *Agrobacterium tumefaciens* LBA4404 [26]. The fungal strains used in this work were: *Phoma medicaginis* strain CT1 and *Colletotrichum trifolii* strain CT2, isolated from INTA alfalfa cultivars and kindly provided by Dr. Ricardo Comerio (Instituto de Microbiología y Zoología Agrícola, Instituto Nacional de Tecnologia Agropecuaria, Argentina).

### Fungus material

Two fungal pathogens, *Phoma medicaginis* strain CT1 and *Colletotrichum trifolii* strain CT2, were grown on PDA (Cat. # B0216605, Britania) plates at room temperature for approximately 7 days before the start of the bioassay. For spore collection, the plates were flooded with sterile distilled water and scraped with a wire loop. Spore concentration was adjusted to 1 × 10^6^ spores/ml for *C. trifolii* CT2 and to 1 × 10^5^ spores/ml for *P. medicaginis* CT1 with sterile distilled water. The fungal strains were maintained through sequential passages in plants.

### Plant material

The *Medicago sativa* plants used were the regenerative clone C2-3, kindly provided by Drs. B. McKersie and S. Bowley (Plant Biotechnology Division, Department of Plant Agriculture, University of Guelph, Canada), and the regenerative clone 432-19-17, previously isolated in our laboratory.

### Bacterial and plant RNA Isolation and cDNA synthesis

Total bacterial (*E. coli*) and plant tissues (roots, steam, leaflets) RNA was extracted by using an RNeasy Mini Kit (Cat. # 74106, Qiagen) following the manufactures’ instructions. Samples of 2 μg total RNA isolated from bacterial cells or plant tissues were reverse-transcribed in a 25 μl reaction using MMLV-RT (Cat. # M1701, Promega). For PCR amplification in bacteria and plants, 1 μl of RT reaction was used. The PCR reactions were carried out in 25 μl with 0.5 μM of each primer [27], using Taq polymerase (Cat#. 11615010, Invitrogen) following the manufactures’ instructions.

### MsSN1 plasmid construction

The *MsSN1* cDNA (GenBank accession number JQ809686) was isolated using primers p1 FW and p2 RV (Additional file 1) designed against the 5′ and 3′ untranslated regions (UTR) of the putative *SN1* gene (GenBank accession number XM_003589066, MTR_1g018640) from *Medicago truncatula*. Full-length cDNA was amplified by PCR and this fragment was cloned into a pCR2.1TOPO vector (Cat. # K4500-01, Invitrogen). The pTOPO-MsSN1 plasmid was digested with Not I (Cat. # R6431, Promega), treated with Klenow, and religated to destroy the polylinker Not I site. The resulting plasmid was named pTOPO-NotI-MsSN1. The sequencing reactions of pTOPO-NotI-MsSN1 were performed at INTA-Argentina (www.inta.gov.ar). The cDNA sequence was named *MsSN1* (*Medicago sativa* snakin-1). To produce recombinant bacteria expressing *MsSN1*, plasmid pSJ33-MsSN1 carrying *MsSN1* was constructed by subcloning the 0.5-kb EcoRI (Cat. # R6011, Promega) fragment from pCR2.1TOPO-MsSN1 into pSJ33 [28], and afterward introduced in *Escherichia coli* for heterologous expression of *MsSN1*. To produce transgenic alfalfa lines overexpressing *MsSN1*, pCR2.1TOPO-NotI-MsSN1 was digested with KpnI (Cat. # R6341, Promega) and XbaI (Cat. # R6181, Promega), and the MsSN1 restriction fragment was cloned into pKANNIBAL vector (GenBank accession number AJ311873). The resulting plasmid was digested with NotI, and the *35SMsSN1* restriction fragment was cloned into pART27 binary vector [29]. The resulting recombinant binary vector containing the *MsSN1* cDNA with its signal peptide under the CaMV 35S promoter was named pART-35S::MsSN1.

### Bioinformatic analysis of MsSN1

*Ms*SN1 sequence of *Medicago sativa* (AFE82743), a peptide composed of 91 amino acids, was used as query to search against all available complete eukaryotic genome databases in NCBI with protein annotation in GenBank. The cut-off to obtain candidate orthologs was 20% of amino acid identity (Additional file 2). Sequences were searched by using BLASTP tools in NCBI and PLAZA databases (http://www.ncbi.nlm.nih.gov/blast; http://bioinformatics.psb.ugent.be/plaza). Protein identity calculations were performed using MatGAT v2.02 [30]. Evolutionary analysis was conducted by using MEGA version 5.0 [31]. Protein sequences were aligned using the ClustalW program. Phylogenetic trees were constructed using the neighbor-joining method with genetic distances computed using the pairwise deletion model and bootstrap analysis of 500 values and root on midpoint (i.e. midpoint of the longest pathway between two clusters of sequences). *In silico* analysis of conserved motifs in putative snakin/GASA proteins, Pfam domains and signal peptides were predicted by using Pfam and Signal-3 L with default parameters, respectively [32,33].

### DNA extraction and sequence analysis of fungal strains

Fungal DNA was extracted as previously described by Moller [34] with modifications suggested by Dr. E.W. Boehm (http://www.eboehm.com/). Briefly, 100 mg of fungal mycelia was scraped from 10-day-old PDA cultures, ground in a 1.5 ml tube with micropestle by adding 500 μl of Lysis Buffer (100 mM Tris pH 8, 10 mM EDTA, 2% SDS, 1% β-Mercaptoethanol, 100 μg/ml proteinase K). Lysate was incubated at 60°C for 60 min. Subsequently, 5 M NaCl was added to a final concentration of 1.4 M and mixed before adding 0.1 vol of CTAB 10% (w/v). The mixture was incubated at 65°C for 10 min. DNA was extracted by adding an equal volume of chloroform:isoamyl alcohol (24:1), incubating for 30 min at 0°C and centrifuging at 14,000 × g for 10 min at 4°C. The aqueous phase was mixed with 0.5 vol of 5 M ammonium acetate, incubated at 0°C for 60 min and centrifuged at 14,000 × g for 1 min. DNA was precipitated from the supernatant by adding 0.55 vol of isopropanol, centrifuged at 14,000 × g for 10 min and washed twice with 70% ethanol. DNA pellet was air-dried and resuspended in 50 μl of TE (10 mM Tris pH 8.0; 1 mM EDTA pH 8.0). The internal transcribed spacer regions 1 & 2 and 5.8S nrDNA (ITS) and partial sequences of TUB genes were amplified and sequenced by Macrogen Inc. (Korea) service (http://www.macrogen.com/) using the primer pairs ITS-1FW and ITS-4RV and Btub2FW and Btub4RV, respectively (Additional file 1). The nucleotide sequences obtained here were deposited in the EMBL Nucleotide Sequence Database, accession numbers: KF846005 for *C. trifolii* TUB, KF846006 for *P. medicaginis* TUB, KF846009 for *C. trifolii* ITS and KF846010 for *P. medicaginis* ITS.

### *In vitro* antimicrobial activity assays

*Escherichia coli* recombinant strains containing the pSJ33 empty vector or pSJ33-MsSN1 were grown overnight at 30°C with shaking (250 rpm) in LB medium supplemented with 1 mM isopropyl-β-d-thiogalactopyranoside in 250-ml Erlenmeyer flasks containing 50 ml of medium. Samples of 25 ml of culture broth (O.D: 0.8) were centrifuged at 5,000 × g at 4°C for 10 min. The pellets were washed three times with physiological solution (0.9% NaCl). Crude cell lysates were achieved by four consecutive cycles of freezing in liquid nitrogen followed by thawing at 37°C. After centrifugation at 14,000 × g at 4°C for 20 min, the supernatant was resuspended in 400 μl of physiological solution. For the analysis of *MsSN1* expression in *E. coli,* total bacterial RNA was extracted by using the RNeasy Mini kit and treated with DNaseI (Cat. # M6101, Promega). cDNA was obtained using random hexamers (Cat. # B070-40, Promega) and AMV Reverse Transcriptase (Cat. # M9004, Promega). For PCR amplification, 1 μl of RT reaction was used. The PCR reactions were carried out in 25 μl with 0.5 μM of each primer according to Setten [27]. PCR conditions comprised: 1 cycle at 94°C for 3 min, 34 cycles of 94°C for 45 s, 56°C for 1 min and 72°C for 1 min. The expression of *MsSN1* was analyzed using primers p3 FW and p4 RV which amplify the complete open reading frame (Additional file 1).

The disk inhibition assays were evaluated as described by Ayub [35], with very slight modifications. Cultures were performed in 125-ml Erlenmeyer flasks containing 25 ml of TY medium [36] or LB medium for rhizobia or *Pseudomonas* and *Agrobacterium*, respectively. Bacteria were incubated overnight at 28°C with shaking (250 rpm). Sterile Whatman No. 1 filter disks (5 mm) impregnated with 5 μl of *Ms*SN1-free (*E. coli* pSJ33) or *Ms*SN1 (*E. coli* pSJ33-MsSN1) extracts were placed on top of bacteria-seeded plates. Zones of inhibition were measured after incubation at 28°C for 24 h. The antifungal activities of the *Ms*SN1 extract were determined according to Kovalskaya [12] by counting germinating and non-germinating fungal spores. The fungal spores of *Phoma medicaginis* var. medicaginis CBS 316.90 from CBS-KNAW Fungal Biodiversity Center (www.cbs.knaw.nl/Collections) were prepared in PDB. For the inhibition assays, spore suspensions of 1×10^5^ spores/ml were used. Each antifungal assay was performed in triplicate.

### Plant transformation

The recombinant binary vector pART-35S::MsSN1 was introduced into *Agrobacterium tumefaciens* LBA 4404 by electroporation, by using the procedure described by Shen & Forde [37]. Petioles of alfalfa clone C2-3 were transformed with pART-35S::MsSN1 via *A. tumefaciens* and cultured *in vitro* as described by D’Halluin [38], with slight modifications (Additional file 3). Petiole tissues were decontaminated by immersing in 70% ethanol for 1 min and then in 2% sodium hypochlorite for 20 min. The petioles were washed 3 times in sterile distilled water. Explants previously injured with a scalpel were inoculated with a bacterial culture for 2 minutes (OD_600 mm_ = 0.5-0.8), and then dried in Whatman filter paper and transferred onto a solid co-cultivation SHK medium [3% sucrose, 0.435% KSO_4_, 2 mg/l 2.4-D, 0.2 mg/l kinetine, 6.5 g/l agar, 20% SHK stock solution (w/v) pH:5.8 (300 mg/l NH_4_H_2_PO_4_, 2.5% KNO_3_, 200 mg/l CaCl_2_.2H_2_O, 400 mg/l MgSO_4_.7H_2_O, 4.3% K_2_SO_4_, 1 mg/l KI, 5 mg/l H_3_BO_3_, 10 mg/l MnSO_4_.H_2_O, 1 mg/l ZnSO_4_.H_2_O, 1 mg/l Na_2_MoO_4_.2H_2_O, 1 mg/l CuSO_4_.5H_2_O, 0.1 mg/l CoCl_2_.6H_2_O, 26.29 mg/l NaFeEDTA.H_2_O, 288 mg/l proline, 53 mg/l thioproline, 200 mg/l myo-inositol, 5 mg/l nicotinic acid, 0.5 mg/l pyridoxine, 5 mg/l thiamine)] containing 100 μM acetosyringone for 2 days at 25°C in the dark. The explants were then washed with 0.5 g/l cefotaxime supplemented with distilled water and transferred to selection/induction medium SHK containing 25 mg/l kanamycin and 400 mg/l cefotaxime with routine transfers to fresh medium every 2 weeks, at 25°C and 16 h light (100 μmoles m^−2^ s^−1^). Somatic embryos were obtained three months later and then transferred to MS rooting medium, composed of Murashige and Skoog Basal Medium (Cat. # M5519, Sigma) diluted 1:2 with water. Transgenic plants were obtained about 6 months after callus induction. The regenerated plantlets were transferred to the greenhouse once they were well rooted. All plants were grown in a greenhouse at 25-20°C day/night temperature and watered daily. Alfalfa transgenic events (named S1, S2 and S3) were propagated by crossing with clone 432-19-17 and by cuttings to increase the number of plants available for biochemical, physiological, and genetic analysis.

### Identification of transgenic plants

DNA was isolated from leaf tissue with the DNeasy plant mini kit (Cat. # 69104, Qiagen). Transgenic plants were first identified by PCR with primers p5 FW and p6 RV (Additional file 1), designed against the 35S promoter and *SN1* regions of recombinant binary vector. The expression of the transgene was corroborated by RT-PCR, using primers p7 FW and p4 RV, designed against the pCR2-1-TOPO-MCS-derived 5′ UTR region and the 5′ UTR of the *MsSN1* gene (Additional file 1). All PCR reactions were performed with Taq (Cat. # 11615010, Invitrogen).

For Southern hybridization analysis, genomic DNA was isolated from leaf tissues of greenhouse-grown plants using the DNeasy Plant Maxi kit (Cat. # 68161, Qiagen) following the manufacture’s indications. DNA was digested with the KpnI restriction enzyme, which cleaves the construct only once. Then, 20 μg of DNA from each sample was digested overnight and blotted after separation on 1% (w/v) agarose gel 1× TAE. The DNA fragments in gels were transferred to a positively charged Nylon membrane (Cat. # 11209272001, Roche). Nylon membranes were crosslinked and then used for hybridization with a DIG-labeled probe. Prehybridization and hybridization was carried out according to the manufacturer’s instructions. The hybridization probe MsSN1digoxigenin-labeled DNA was generated by PCR by using the PCR DIG probe synthesis kit (Cat. # 11573152910, Roche), using primers p8 FW and p9 RV and then used as a probe (Additional file 1). PCR amplification was performed under standard conditions (25 μl volume using 0.8 μM of each primer, 1X PCR buffer, 0.2 mM each dNTP, 2 mM MgCl_2_ and 20 ng of template) with a program of 34 cycles of 94°C for 1 min, 50°C for 30 s and 72°C for 2.5 min and a final cycle of 72°C for 10 min.

### Real-time quantitative RT-PCR (RT-qPCR)

For RT-qPCR, PCR amplification was performed with 5 μl of RT (1:5 diluted) per reaction, by using 1 U iQ SYBR green Supermix (Cat. # 170–8880, Bio-Rad) and 0.2 mM primers, with the iCycler iQ system. Primers for the real-time qPCR were p10 FW and p11 RV (Additional file 1). qPCR conditions comprised: 1 cycle at 94°C for 5 min, 34 cycles of 94°C for 45 s, 59.1°C for 30 s, and 72°C for 30 s. At each cycle, accumulation of PCR products was detected. The amplification fragment was sequenced and found to be identical (100% nucleotide identity) to the MsSN1 gene. The expression level of MsSN1 was normalized using aspartate aminotransferase (ATT) (AAB46610) as a housekeeping gene, using primers p12 FW and p13 RV (Additional file 1). The efficiency of primer binding was determined by linear regression by plotting the cycle threshold value versus the log of the cDNA dilution [39]. RT-qPCR experiments were performed two or three times with independent RNA samples (biological replicates). For each biological replicate the qPCR reactions were carried out in duplicate.

### Bioassays. *In vitro* challenges

Assays were performed in healthy 15 day-old non-fumigated leaflets. Leaflets were decontaminated washing them in flasks with sterile water with Tween-20 (0.01%) for 10 min. After three washes with sterile water, the leaflets were transferred to a 5 % hypochlorite solution for 5 min. Finally, leaflets were washed three times with sterile water and transferred to agar-water petri dishes. Plates were maintained in growth chambers programmed for 16 h light at 23°C and 8 h dark at 20°C. Leaflets were inoculated by putting 5 μl of *P. medicaginis* CT1 or *C. trifolii* CT2 spore solution per leaflet. Infection evolution was observed and documented by photos and the following damage score was generated to quantify injuries: 1. Healthy leaflet, 2. Countable injuries, 3. Uncountable injuries, 4. Chlorosis, 5. Completely damaged.

### Bioassays. *In vivo* challenges

Alfalfa transgenic seeds were obtained by crossing transgenic plants S1, S2 and S3 with 432-19-17 genotype plants. Transgenic and wild type seeds were treated with sulfuric acid for 10 minutes, washed three times with sterile water and placed in petri dishes with 1% agar water at 16 h of light (100 μmoles/m^2^s) and 25°C. Germinated plantlets were transferred to MS 0.5X flasks and incubated at 25°C with 16 h photoperiod for 1 month, after which plants were transferred to 1:1 vermiculite:perlite and maintained in magenta vessels (SIGMA) to conserve the humidity. Two-month-old plants were inoculated with *P. medicaginis* CT1 by spraying spore suspension to all aerial tissues. Percentage of diseased leaflet was analyzed 30-day post-inoculation and the number of plants with regrowth and the percentage of highly defoliated plants was counted 60-days post-inoculation. As an additional severity parameter of plant disease, the percentage of vigor affected plants (i.e. with visual signs of turgidity loss compared with non-inoculated plants) was estimated. All experiments were carried out with at least 10 independent plants per treatment and with at least 5 non inoculated wild type plants used as control.

### Evaluation of bacterial root colonization

For the symbiosis assay, wild type and transgenic alfalfa seedlings were grown in 100% vermiculite and daily irrigated with the minimal medium called “INTA13 without nitrogen” [27]. 10-days-old plants were inoculated with an early stationary-phase culture of rhizobial suspension (*Sinorhizobium meliloti* BL225C). After a month, the plants were harvested and the number of pink nodules was analyzed. Non-inoculated plants were used as controls. Three replicates were analyzed for each treatment. The *P. fluorescens* Pf-5 colonization assay was evaluated according to Sanchez [40], with two slight modifications: alfalfa was grown in INTA13 supplemented with Ca (NO_3_)_2_ [27] and 0.5 NE2 medium plus sodium octanoate (0.25% w/v) was used to estimate the colony forming units [28].

### Statistical analysis

Biological measurements were repeated at least three times with at least 10 different plants. Significant differences between treatments were calculated using Student’s t-test. qPCR experiments were independently performed three times, with comparable results. The three PCR reactions were carried out in duplicate. Significant differences were calculated using ANOVA followed by Tukey test.

## Results and discussion

### Identification and evolutionary analysis of MsSN1

**Figure 1:**
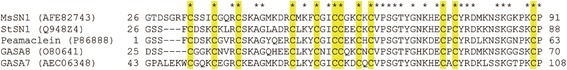
**Alignment of residues 26–91 of** ***Ms*** **SN1 from alfalfa with the corresponding regions of snakin/GASA proteins from potato (** ***St*** **SN1), Arabidopsis (GASA7 and GASA8) and Peach (Peamaclein) revealing 12 cysteine residues in conserved positions within a conserved C-terminal region.** These cysteine residues are shown in yellow.

To analyze the evolution and function of *MsSN1*, a 276-bp cDNA fragment was isolated from roots by RT-PCR, using specific primers designed for a putative snakin-1 peptide predicted from the genome of the model legume *Medicago truncatula* (*Mt*SN1, MTR_1g018640). The PCR product was cloned into a pCR2.1TOPO vector and sequenced (JQ517286). This cDNA fragment shares 98% identity with the putative snakin-1 peptide from *Medicago truncatula* (XP_003589114), hence it was named *MsSN1* (*Medicago sativa* snakin-1). Like potato snakin-1 peptide (*St*SN1), *Ms*SN1 has a putative signal peptide of 25 amino acids and possesses a snakin/GASA domain (Pfam02704) containing 12 cysteine residues in conserved positions within a conserved C-terminal region (Figure 1).

**Figure 2:**
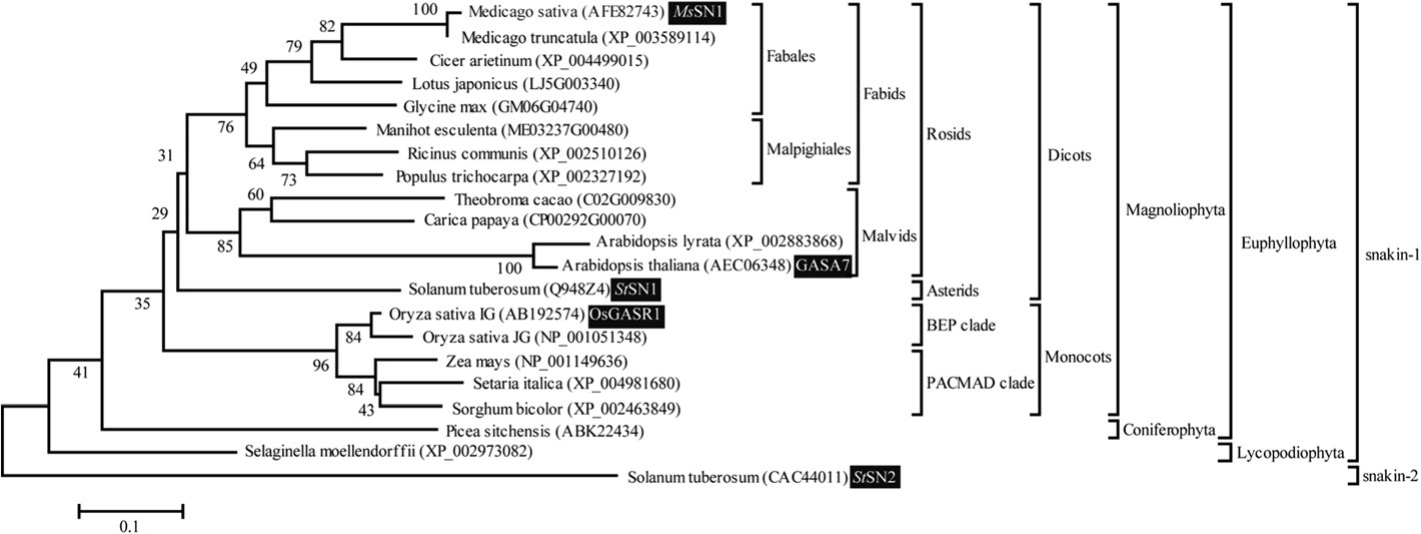
**Phylogenetic tree of plant SN1 protein sequences using the neighbor-joining method and root on midpoint.** Bootstrap percentages are indicated at the branch points. The current classification of plants is found on the right. Tree topology obtained using NJ method, Minimum evolution and Maximum parsimony methods were identical. The *Ms*SN1 peptide characterized in this work and other snakin/GASA peptides described in previous experimental studies are boxed.

To perform a phylogenetic analysis of SN1, the evolutionary study was restricted to well-characterized sequenced species of plants using proteins with high amino acid identity (>20%). Using this strict criterion, complete congruence (i.e. same topology) between SN1 and vascular plant trees was observed (Figure 2), suggesting that plant SN1 was acquired by vertical transfer [41]. Since our phylogenetic analysis of SN1 was consistent with rRNA data, orthologous SN1 assignment is possible in any vascular plant. Thus, our evolutionary study suggests that *Ms*SN1 from *Medicago sativa* (JQ517286) presented in this work is indeed the ortholog of the *St*SN1 from *Solanum tuberosum* (Q948Z4), the gibberellin-stimulated transcript 1 (OsGASR1) from *Oryza sativa* (AB192574) and the GASA7 from *Arabidopsis thaliana* (AEC06348) previously described [3,42,43], thus offering a starting point for experimental data integration to in-depth understanding of SN1 function in plants. It is important to note that orthologous identification of snakin-like peptides could not be predicted by comparing their amino acid identity with snakin/GASA-related proteins such as Arabidopsis GASAs (Additional file 4). Therefore, assessment of the correct orthology of snakin/GASA genes requires rigorous phylogenetic analysis.

In order to support the vertical origin of higher plant SN1, the *SN1* gene is present in the primitive vascular plant *Selaginella moellendorffii* (Figure 2). In addition, an exhaustive phylogenetic analysis of proteins within genome databases failed to find orthologous genes of SN1 in the ancestral moss *Physcomitrella patens*, and in green algae species belonging to the phylum *Chorophyta* such as *Ostreococcus lucimarinus*, *Micromonas* sp. RCC299, *Volvox carteri* and *Chlamydomonas reinhardtii*. In agreement with this finding, proteins containing the snakin/GASA domain are widely distributed in land plants but completely absent in moss and green algae (data not shown), suggesting that *SN1* is an ancestral gene that appeared in the vascular lineage after the vascular/bryophyte separation. This is not surprising considering that the pioneering ancestors of land plants that dominated terrestrial environments had to confront dramatic stresses, including the infection produced by an extreme diversity of microbial soil communities, selecting a number of genetic innovations [44]. Therefore, the emergence of SN1 could be an adaptation of ancestral plants to land.

### Analysis of the *in vitro* antimicrobial activity of MsSN1

**Figure 3:**
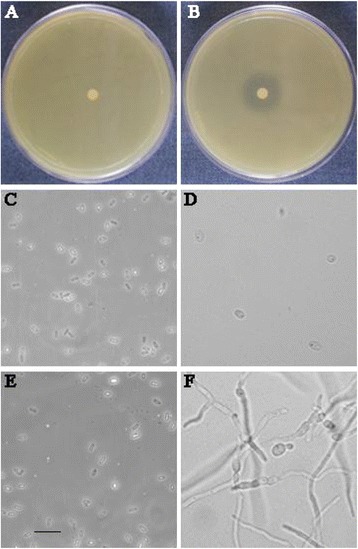
**Analysis of the** ***in vitro*** **antimicrobial activity of** ***Ms*** **SN1.** *Agrobacterium* growth **(A-B)** and *Phoma* spore germination (1 × 10^5^ spores/ml) **(C-F)** inhibition assays using *Ms*SN1-free **(A, C and D)** or *Ms*SN1 extracts **(B, E and F)**. Spore germination was examined immediately **(C and E)** and after 16 h **(D and F)** of incubation with the extracts. Bar = 50 μm.

Regarding the *in vitro* characterization of *Ms*SN1 antimicrobial activity, the *MsSN1* gene was expressed in *E. coli*. The expression of *MsSN1* in the transformed *E. coli* cells was confirmed by RT-PCR analysis (Additional file 5). The antimicrobial activity of *Ms*SN1-free or *Ms*SN1 extracts was tested against the bacterial and fungal alfalfa pathogens *Agrobacterium tumefaciens* LBA4404 and *Phoma medicaginis* var. medicaginis CBS 316.90, respectively. Antimicrobial activity of the *Ms*SN1-free extract was undetectable, whereas the *Ms*SN1 extract showed a high inhibitory activity against bacterial growth (Figure 3A-B) and fungal spore germination (Figure 3C-F). As previously described for other members of snakin/GASA peptides [3,4], the antimicrobial activity of *Ms*SN1 against organisms belonging to different domains, at least Bacteria and Eukarya, is congruent with its function as a component of the non-specific immune system of plants.

### Analysis of MsSN1 expression

**Figure 4:**
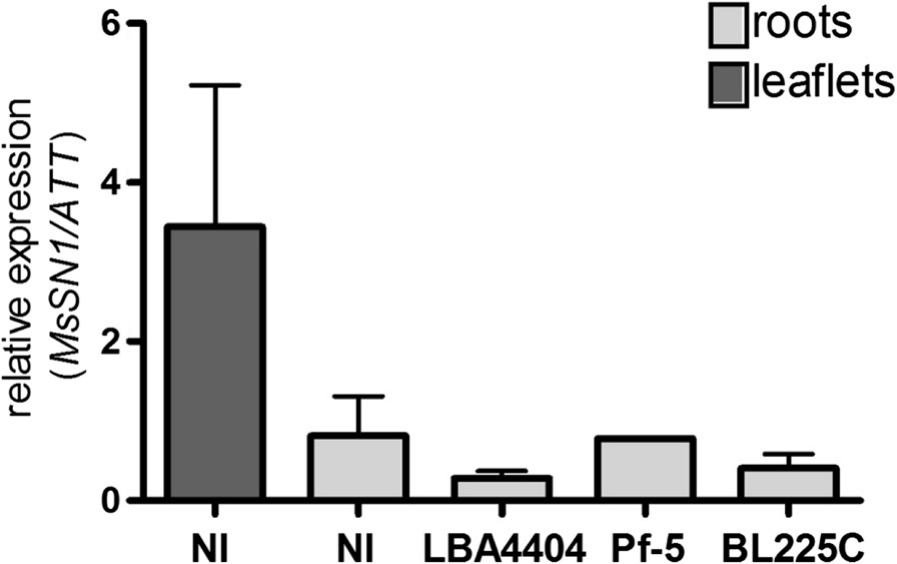
**Quantitative analysis of the expression of the** ***MsSN1*** **gene in wild type alfalfa plants under microbe stress conditions.** Real-time RT-PCR studies for *MsSN1* expression in non-inoculated leaves and roots (NI) or in roots exposed to *Agrobacterium tumefaciens* LBA4404, *Pseudomonas fluorescens* Pf-5 and *Sinorhizobium meliloti* BL225C for 24 hs.

The expression pattern of the *MsSN1* gene was analyzed by RT-PCR, to explore the function of *Ms*SN1 in wild type alfalfa plants. *MsSN1* expression was detected in all tissues analyzed, including roots, stems, leaves and young floral buds (Additional file 6). RT-qPCR assays were performed to evaluate whether the expression levels of *MsSN1* is modified by exposure to microorganisms. *MsSN1* transcript levels were about two-fold higher in leaves than in roots when plants were grown under control conditions (Figure 4). Interestingly, *MsSN1* levels did not change in response to biotic stimuli, such as the pathogenic bacterium *A. tumefaciens* LBA4404, the symbiotic strain *Sinorhizobium meliloti* BL225C and the free-living strain *Pseudomonas fluorescens* Pf-5 (Figure 4). In concordance with the characterization of *StSN1* expression in potato [3], the expression pattern of *MsSN1* suggests that *SN1* is a component of the constitutive defense barrier.

### Molecular characterization of transgenic alfalfa plants overexpressing MsSN1

**Figure 5:**
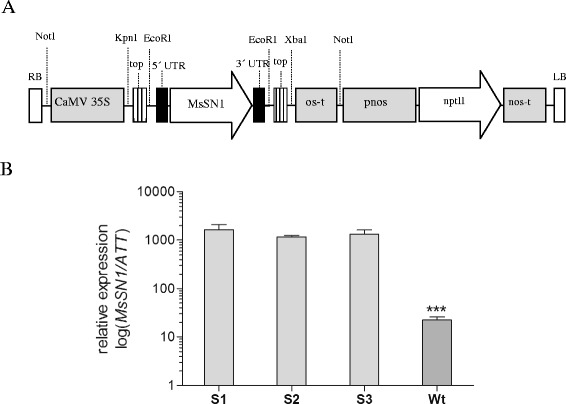
**Characterization of transgenic alfalfa lines overexpressing** ***MsSN1*** **. (A)** Schematic representation of the T-DNA region of binary vector pART-35S::MsSN1 containing the *MsSN1* gene under the CaMV 35S promoter. Relevant restriction enzymes used in plasmid construction and Southern Blot analysis are shown. RB: right border; CaMV 35S: promoter; Topo: region derived from pCR2.1-TOPO vector; UTRs: untranslated regions derived from the native *MsSN1* gene; os-t: octopine synthase terminator; pnos-nptII-nos-t: kanamycin cassette (where, pnos and nos-t are nopaline synthase promoter and terminator, respectively); LB: left border. **(B)** Real-Time RT-PCR assays of alfalfa transgenic lines (S1-S3) and control untransformed plants (wt). All values are log means ± SEM (n = 3). Asterisks indicate a statistically significant difference (Tukey: ***p < 0.001).

In order to investigate the antimicrobial activity of *MsSN1* against alfalfa fungal pathogens, transgenic alfalfa plants were generated. The *MsSN1* gene was placed into a constitutive expression cassette under the control of the CaMV 35S promoter (Figure 5A), and introduced in alfalfa plants of the regenerable clone C2-3 by *Agrobacterium tumefaciens*-mediated transformation (Additional file 3). PCR assays (Additional file 7A) and Southern Blot analyses (Additional file 7B) were used to identify three independent transgenic alfalfa lines, named S1, S2 and S3, harboring the CaMV35S:MsSN1 construct. Evolutionary analyses showed that *Medicago truncatula* and *Lotus japonicus* genomes have only one locus per genome of the SN1 gene, MTR_1g018640 and LJ5G003340, respectively. Consistent with this, as well as with the remarkably conserved genome structure among legumes [45] and the tetrasomic inheritance and outcrossing nature of alfalfa [46], the non-transformed alfalfa plants showed the presence of three copies, probably alleles, of the *MsSN1* gene (Additional file 7B). As expected, transgenic plants showed extra and differential bands on Southern blot hybridizations, suggesting the incorporation of the transgene in different regions of the alfalfa genome (Additional file 7B). The three events showed stable inheritance of the transgene (data not shown). Expression of the transgene was confirmed by RT-PCR analyses using specific primers designed for the chimeric 5′ UTR (Additional file 7C). Individual lines were also analyzed by RT-qPCR assays for *MsSN1* expression. Transgenic lines showed that the expression level of total *MsSN1* was 100-fold higher than wild type endogenous expression (Figure 5B), suggesting that the three transgenic lines were good candidates to evaluate the effects of overexpressing *MsSN1*.

Similar to transgenic lines from potato, wheat and tomato [6,10,11], overexpression of *MsSN1* did not appear to affect negatively the general phenotype in transgenic alfalfa plants, e.g. there was no reduction in the plant vigor in transgenic plants compared to wild type plants (Additional file 8). Therefore, transgenic alfalfa plants appear to be a suitable platform to study the role and biotechnological potential of *MsSN1*. Unfortunately, no *MsSN1* reduced expression or silenced alfalfa plants were obtained (data not shown). Similar results were reported by Meiyalaghan [8] where the authors found no regenerant plants using antisense vectors expressing GSL1 (*StSN1*) and GSL2 (*SN2*) under their endogenous promoters regulation. These results probably indicate that *MsSN1* silencing had detrimental effects on transformation or regeneration efficiency. In concordance with this observation, *SN1* silencing drastically affects the cell division and primary metabolism of potato plants [14].

### Molecular characterization of two highly virulent alfalfa fungal strains

Anthracnose and spring leaf spot, caused by the fungal pathogens *Colletotrichum trifolii* and *Phoma medicaginis*, respectively, are the most destructive infections of alfalfa worldwide. In the analysis of alfalfa resistance to these fungal diseases, there is usually a tradeoff between virulence and characterization of these pathogenic strains. Generally, long-term successive passages of archetype strains *in vitro* attenuate the virulence of these pathogens *in vivo,* while pathogens freshly isolated present a high virulence phenotype but their taxonomic position is commonly speculated based on disease symptoms or microscopy observations. In order to bypass this constraint, we used two highly virulent alfalfa fungal strains from alfalfa breeding programs, maintained through sequential plant infections and characterized through amplification, sequencing and phylogenetic analyses of conserved genes and intergenic regions. The evolutionary analyses of ITS region and TUB gene showed that these isolates belong to *Colletotrichum* and *Phoma* (Additional file 9). In fact, these DNA fragments from virulent strains (KF846005, KF846006, KF846009 and KF846010) share 100% nucleotide identity with the ITS region and TUB genes from *C. trifolii* CBS 158.83 (KF178478 and KF178599) and *P. medicaginis* CBS 316.90 (GU237828 and GU237630), respectively. In accordance with these data, these isolates were named *Colletotrichum trifolii* strain CT2 and *Phoma medicaginis* strain CT1. Unlike the well-characterized fungal strains *C. trifolii* CBS 158.83 and *P. medicaginis* CBS 316.90, strains *C. trifolii* CT2 and *P. medicaginis* CT1 are highly virulent on alfalfa and infect a wide range of commercial cultivars as well as the alfalfa clones used in this work, C2-3 and 432-19-17. This confirms that the form of conservation of fungal pathogens is essential to maintain their alfalfa virulence, and therefore, to study the genetic basis of resistance to fungi in alfalfa.

### Antifungal *in vitro* activity of MsSN1-overexpressing transgenic plants

**Figure 6:**
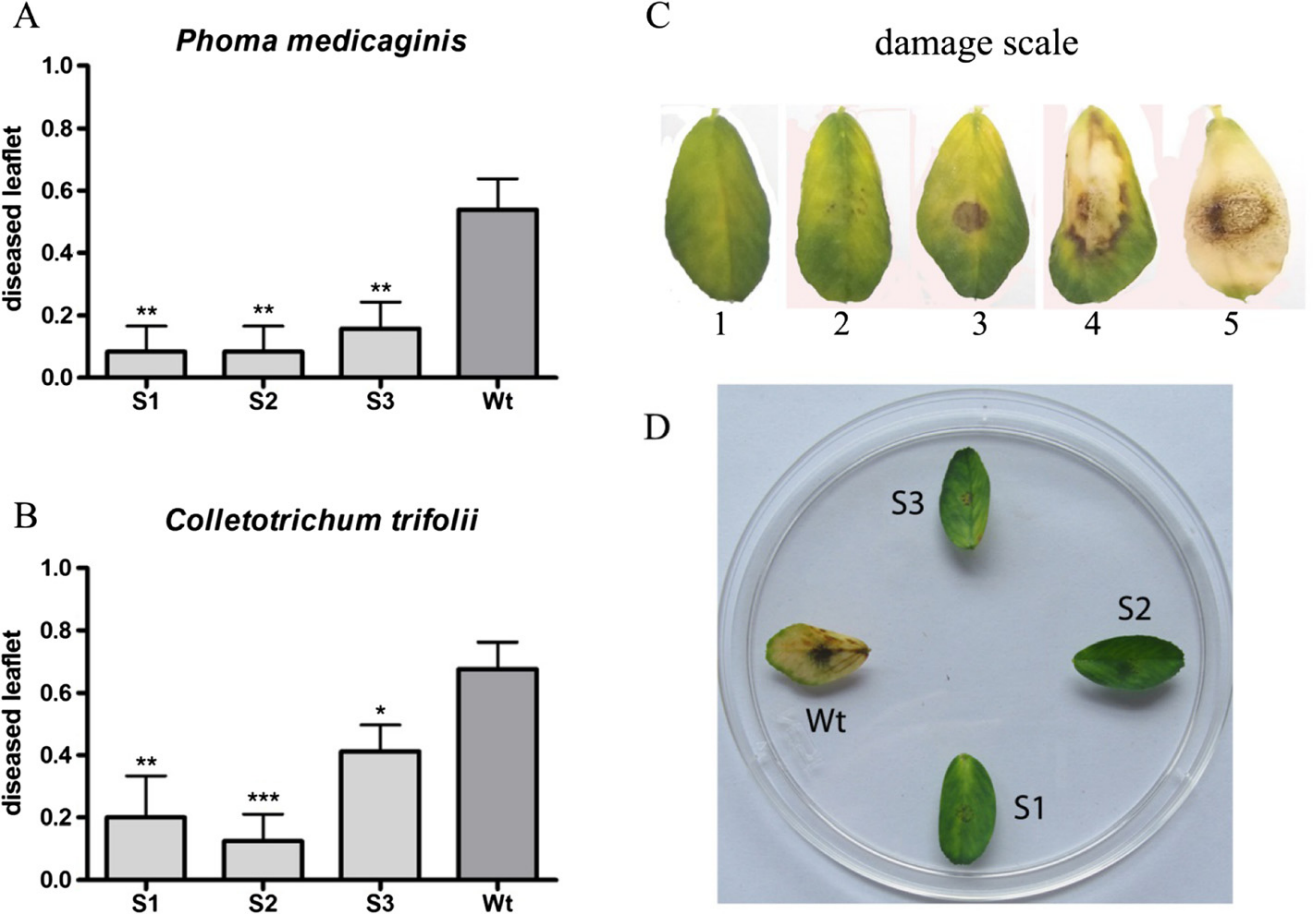
**Antifungal** ***in vitro*** **activity of** ***MsSN1*** **overexpressing transgenic plants. (A)** Diseased leaflets related to leaflets inoculated with *Phoma* (#diseased leaflet/#total leaflet)*.* **(B)** Diseased leaflets related to leaflets inoculated with *Colletotrichum* (#diseased leaflet/#total leaflet). **(C)** Damage score: 1. Healthy leaflet, 2. Countable injuries, 3. Uncountable injuries, 4. Chlorosis, 5. completely damaged. **(D)** Representative *Phoma assay*. Wt: wild type. S1-S3: *Ms*SN1 transgenic plants. All values are log means ± SEM (n = 10–30). Asterisks indicate a statistically significant difference (Turkey: *p < 0.5; **p < 0.01 ***p < 0.001). Leaflets were considered diseased when they showed symptoms 3, 4 or 5 in the damage score. Disease severity was estimated from scoring 30 to 60 detached leaflets from three individual plants.

As a first step to investigate the antifungal activity of transgenic alfalfa lines, *in vitro* challenge assays were performed. Detached leaflets were inoculated with a spore suspension of two virulent and molecularly characterized alfalfa fungal strains *P. medicaginis* CT1 and *C. trifolii* CT2 incubating the leaves in petri plates for 7 days (Figure 6A-B). Disease severity was estimated by visually scoring disease symptoms in infected leaflets as a percentage of leaflets showing disease lesions (Figure 6C). The three transgenic lines (S1- S3) showed significant lower percentage of diseased leaflets than wild type plants when challenged with both *P. medicaginis* CT1 (Figure 6A) and *C. trifolii* CT2 virulent strains (Figure 6B), suggesting an antimicrobial function of *MsSN1* under biotic stress conditions. Moreover, this small-scale study could facilitate rapid visual screening of a large number of transgenic events (Figure 6D), saving time, reducing cost, and speeding up the introgression of this antimicrobial transgene into commercial varieties, especially in outcrossing species such as alfalfa [47].

### Antifungal *in vivo* activity of MsSN1-overexpressing transgenic plants

The three transgenic alfalfa lines (S1-S3) were chosen for progeny analysis of their potentially enhanced biotic resistance. The clone 432-19-17 was used as a pollen donor in a sexual cross with the transgenic lines to avoid the common inbreeding depression of progeny from self-crossed alfalfa plants. The presence and expression of the transgene in leaves of the progeny plants were assayed by PCR and RT-qPCR analyses, respectively (data not shown). *In vivo* assays for antifungal activity against *P. medicaginis* CT1 were carried out spraying a spore solution to transgenic and wild type plants. Plant disease severity was estimated by evaluating agronomic parameters for two months. Wild type plants manifested all the symptoms of the disease [48,49], including dark brown lesions on the stems, dark brown lesions on the leaflets that begin with specific dark color injuries, wilting leaves, chlorosis, and defoliation. Transgenic lines presented scarce symptoms and usually looked like control plants (data not shown).

**Figure 7:**
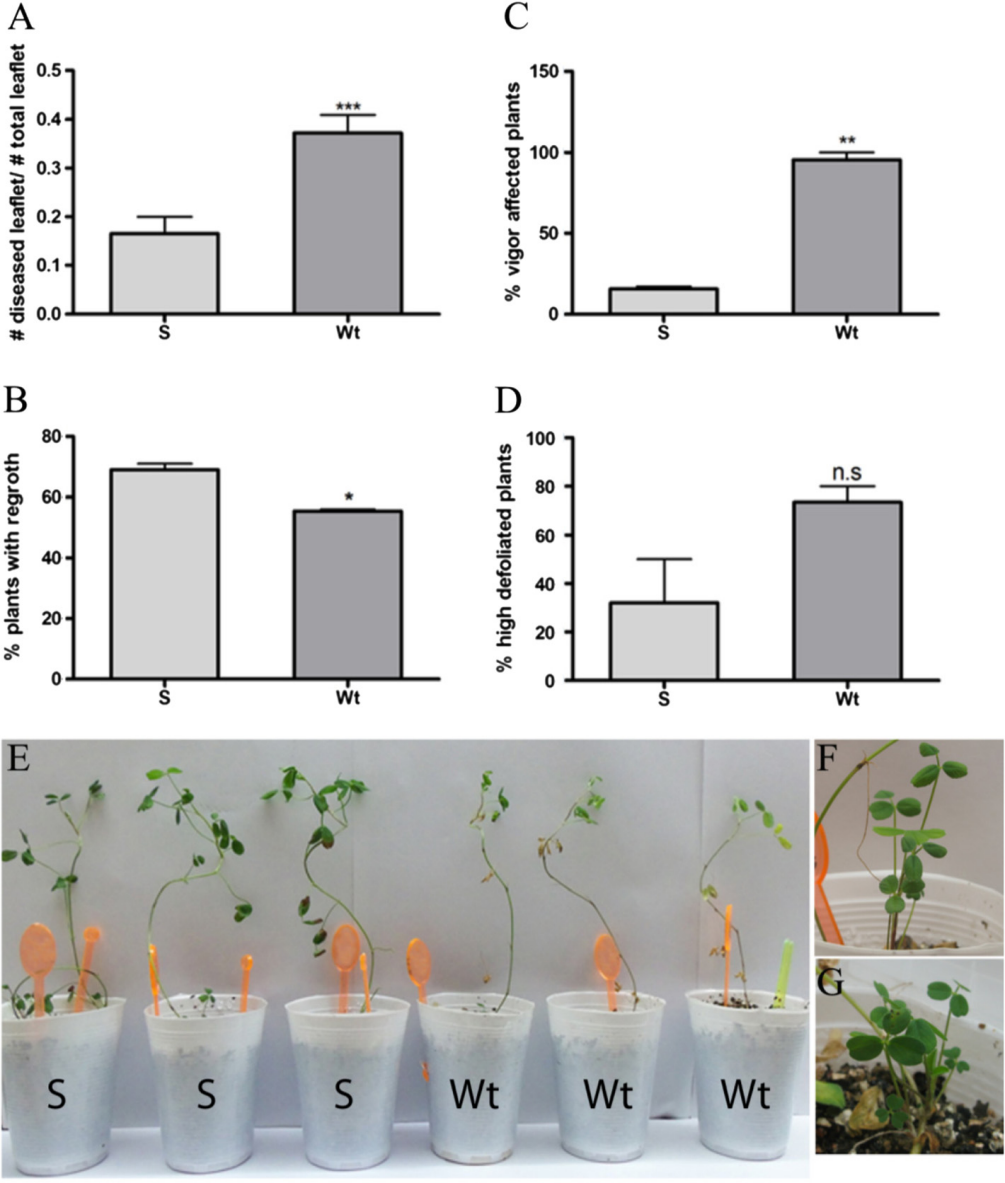
***In vivo*** **characterization of** ***MsSN1*** **overexpressing transgenic plants for resistance to** ***Phoma medicaginis*** **CT1. (A)** Number of diseased leaflets related to total number of leaflet in 30-day-old inoculated plants. **(B)** Percentage of two-month-old inoculated plants with regrowth. **(C)** Percentage of vigor affected two-month-old inoculated plants. **(D)** Percentage of highly defoliated two-month-old inoculated plants (highly defoliated > 3 leaflets detached). **(E)** Representative photo comparing transgenic (S) with wild type (Wt) 30-day-old inoculated plants. **(F)** Detail of regrowth. **(G)** Detail of symptomatic regrowth. S: *MsSN1*-overexpressing plants. Wt: wild type. All values are means ± SEM (n =15-25). N.S: non-significant; *p < 0.05; **p < 0.01; ***p < 0.001, t-test.

Firstly, leaflet disease was estimated by visually scoring disease symptoms in infected plants. One month post-infection, the percentage of diseased leaflets was significantly lower in transgenic lines than in wild type plants (Figure 7A). Two months post-inoculation, disease severity was analyzed by evaluating the vigor of the plants, the number of plants with regrowth and the percentage of highly defoliated plants. Transgenic plants showed significantly higher levels of regrowth than wild type plants (Figure 7B, F and G). In fact, disease symptoms were present in wild type regrowth but not in transgenic plants (Figure 7G). Overexpression of *MsSN1* in alfalfa improves plant vigor under fungal stress conditions (Figure 7C). In addition, wild type plants showed more defoliation than transgenic plants but the differences were not statistically significant (Figure 7D). Altogether, transgenic plants showed a better performance against *Phoma* infection than wild type plants, suggesting an antifungal activity of *Ms*SN1 *in vivo*.

### Coevolution evidences of MsSN1 and rhizobia

Similar to observations on other effector molecules of innate immunity [50], constitutive expression of *MsSN1* could provide alfalfa a selective advantage over the pathogens during the early stages of microbial infection. However, it can also have adverse effects on beneficial microorganisms such as nitrogen-fixing symbiotic bacteria. To investigate this apparent paradox, the *in vitro* antimicrobial activity of *Ms*SN1 against nine rhizobacterial strains isolated from different legume plants and soil were analyzed. All rhizobacterias isolated from alfalfa nodules (*Sinorhizobium meliloti* BL225C, *Sinorhizobium meliloti* SM11 and *Rhizobium* sp. Or 191) showed no inhibition against the *Ms*SN1 extract (Table 1). In contrast, the symbiotic nitrogen fixation strains isolated from other legume species, such as medick (*Sinorhizobium medicae* WSM419), soybean (*Sinorhizobium fredii* USDA 257 and *Bradyrhizobium japonicum* USDA110), bean (*Rhizobium etli* CFN 42) and *Lotus* spp. (*Mesorhizobium loti* MAFF303099), were susceptible to the *Ms*SN1extract (Table 1). These results suggest that alfalfa and their symbiotic bacteria may have coevolved.

**Table 1:** **Antimicrobial activity of alfalfa snakin-1 extract on rhizobial strains**

**Strain**	**Family**	**Source of the strain**	**Zone of inhibition (mm)**
*Bradyrhizobium japonicum* USDA110	Bradyrhizobiaceae	Soybean	1.38 ± 0.13
*Mesorhizobium loti* MAFF303099	Phyllobacteriaceae	Lotus spp.	1.50 + 0.30
Rhizobium sp. Or 191	Rhizobiaceae	Alfalfa	0
Rhizobium etli CFN 42	Rhizobiaceae	Bean	1.18 + 0.09
*Sinorhizobium meliloti* BL225C	Rhizobiaceae	Alfalfa	0
*Sinorhizobium meliloti* SM11	Rhizobiaceae	Alfalfa	0
*Sinorhizobium medicae* WSM419	Rhizobiaceae	Medick	1.26 ± 0.06
*Sinorhizobium fredii* USDA 257	Rhizobiaceae	Soybean	1.13 ± 0.13

There is a fine line between bacterial symbiosis and chronic infection. While one is beneficial the other is detrimental. Recent findings suggest that both share mechanisms for sidestepping host defenses [50]. Legumes evolved rapidly and shortly after their origin, and nodulation most likely evolved several times during their divergence [51]. These plants have symbiotic nitrogen-fixing bacteria living in root nodule compartments that also contain antimicrobial compounds. To avoid infection with phytopathogenic bacteria, nitrogen-fixing rhizobial bacteria and leguminous plants have developed complex signal exchange mechanisms that allow a specific bacterial strain to induce its specific host plant species to form invasion structures through which the bacteria can enter the plant root [52]. The evidence of co-evolution presented here suggested that *Ms*SN1 peptide could be part of the battery of compounds involved in discriminating the symbionts from other microbes, so as to allow the beneficial infection and inhibit colonization of potentially pathogenic microbes.

**Figure 8:**
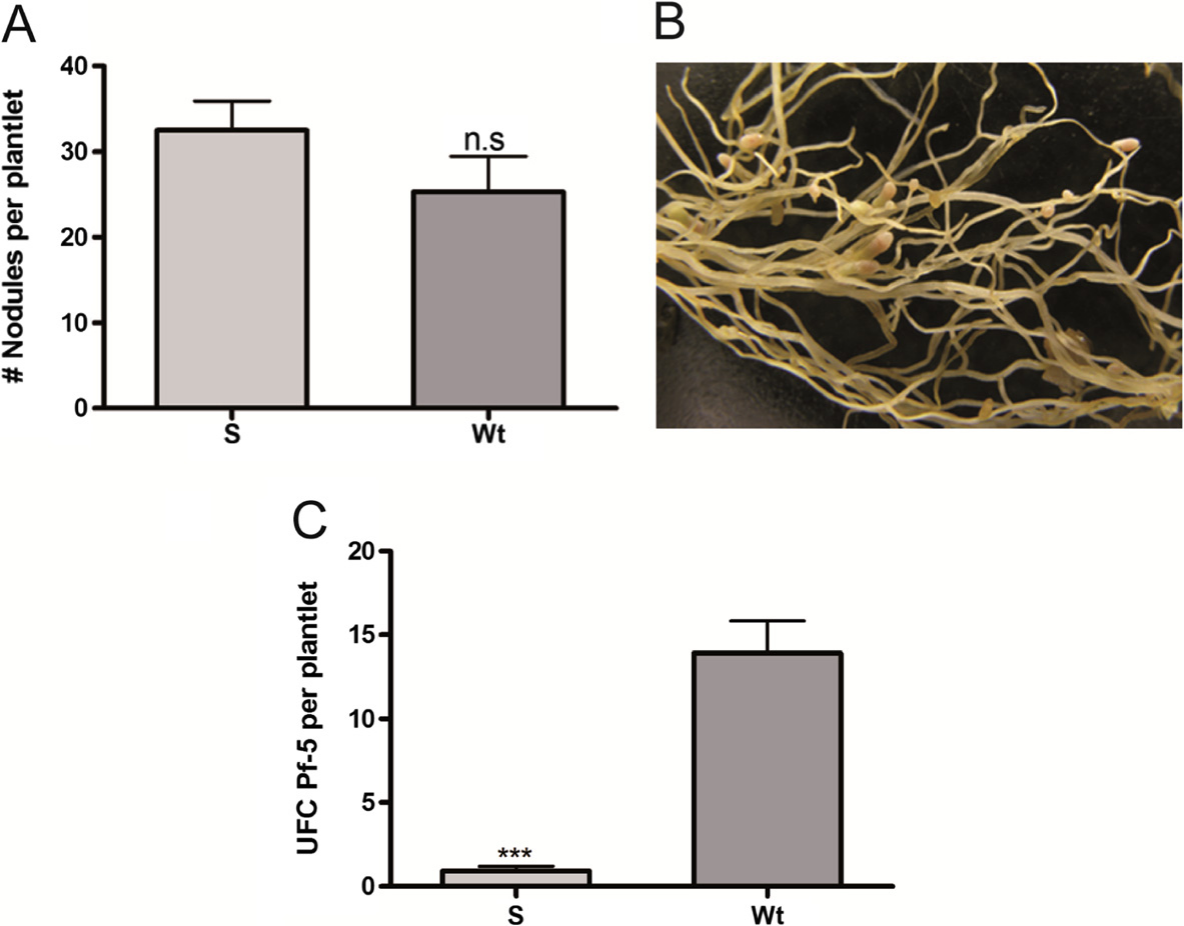
**Bacterial colonization of** ***MsSN1*** **-overexpressing transgenic plants.** Analysis of alfalfa-*Sinorhizobium meliloti* BL225C interactions: number of nodules in two-month-old alfalfa plants. **(A)** Photo of nodulated root. **(B)** Study of alfalfa colonization by *Pseudomonas fluorescens* Pf-5. **(C)** *MsSN1*-overexpressing plants. Wt: wild type. All values are means ± SEM (n =20). N.S: non-significant, ***p < 0.001, t-test.

In concordance with the hypothesis of plant-microbe coevolution, *MsSN1*-overexpressing transgenic plants showed nodule production of *S. meliloti* BL225C comparable with that of the wild type plants (Figure 8A-B), suggesting that *Ms*SN1 does not affect plant infection when nitrogen-fixing bacterial strains have an alfalfa origin. Then, to explore the effect of *Ms*SN1 on free living strains of rhizobacteria as *Pseudomonas*, the antimicrobial activity of *Ms*SN1 on *P. fluorescens* Pf-5 isolated from soil samples was studied. Interestingly, *P. fluorescens* Pf-5 showed hypersensitivity to the *Ms*SN1extract (Zone of inhibition = 1.76 ± 0.06 mm). In agreement with the *in vitro* activity of *Ms*SN1, *P. fluorescens* Pf-5 showed differential proliferation in the rhizosphere of wild type and transgenic lines of alfalfa 30 days after inoculation, suggesting an increased antibacterial activity in *MsSN1*-overexpressing plants (Figure 8C). These results show additional *in vitro* and *in vivo* evidence of *Ms*SN1 as a part of the innate immune response and the wide range of activity of *Ms*SN1.

## Conclusions

Previous studies and the results reported here show that snakin peptides have important and ancestral roles in land plant innate immunity. Interestingly, *MsSN1*-overexpressing alfalfa transgenic plants show increased antimicrobial activity against virulent fungal strains without altering the nitrogen-fixing symbiosis, opening the way to the production of effective alfalfa transgenic cultivars for biotic stress resistance. In this work, data also suggest a coevolutionary process, in which alfalfa exerts a selection pressure for resistance to *Ms*SN1 on rhizobial bacteria.

## Additional files

Additional file 1
**Table of primers used in this work.**
Additional file 2
**Similarity of the product of** ***MsSN1*** **gene to other snakin/GASA proteins from plants.**
Additional file 3
**Alfalfa transformation.**
Additional file 4
**Identity of the product of** ***MsSN1*** **gene to other snakin/GASA proteins from Arabidopsis.**
Additional file 5
**RT-PCR studies of** ***MsSN1*** **gene expression in** ***E. coli*** **pSJ33 (1) and** ***E. coli*** **pSJ33-MsSN (2) strains.**
Additional file 6
**RT-PCR assays showing** ***MsSN1*** **expression in young floral buds (A-B), leaves (C-D), stems (E-F) and roots (G-H).**
Additional file 7
**Molecular characterization of transgenic alfalfa lines overexpressing** ***MsSN1.***
Additional file 8
**Vigor phenotype of transgenic (S1) and wild type (Wt) alfalfa plants.**
Additional file 9
**Phylogenetic analysis of fungal strains isolated from alfalfa.**
