# Supplementary material for: Alfalfa snakin-1 prevents fungal colonization and probably coevolved with rhizobia
Source: BMC Plant Biol. 2014 Sep 17;14:248. doi: 10.1186/s12870-014-0248-9 (PMC4177055; doi:10.1186/s12870-014-0248-9)
Supplement: Additional file 1 — Table of primers used in this work. [file 12870_2014_248_MOESM1_ESM.doc]

**Additional file 1**. Table of primers used in this work

| **Primer name** | **Sequence 5´-3´** |
| --- | --- |
| p1 FW | ggcttccttagtgagttttcctt |
| P2 RV | gagaatgggaataaatcagggtc |
| ITS-1FW | TCCGTAGGTGAACCTGCGG |
| ITS-4RV | TCCTCCGCTTATTGATATGC |
| Btub2FW | GTBCACCTYCARACCGGYCARTG |
| Btub4RV | CCRGAYTGRCCRAARACRAAGTTGTC |
| p3 FW | atgaagccagcatttgcagc |
| p4 RV | TTAAGGGCATTTTGGCTTTCC |
| p5 FW | ATCTCCACTGACGTAAGGGA |
| p6 RV | ACCCTGATTTATTCCCATTCTC |
| P7 FW | TCGGATCCACTAGTAACGGCC |
| p4 RV | TTAAGGGCATTTTGGCTTTCC |
| p8 FW | ATTTGCAGCTATGTTACTTGTGT |
| p9 RV | CATTTTGGCTTTCCCTTAGAG |
| p10 FW | TATGTTCGAATGTCCATTGCT |
| p11 RV | TTCCTGATGGCACACACTTAC |
| p12 FW | CAATTTCGCATCTCATTAAGATCG |
| p13 RV | ACCACATCCCAAATAAATAAGATTCTAAC |
