# Supplementary material for: Alfalfa snakin-1 prevents fungal colonization and probably coevolved with rhizobia
Source: BMC Plant Biol. 2014 Sep 17;14:248. doi: 10.1186/s12870-014-0248-9 (PMC4177055; doi:10.1186/s12870-014-0248-9)
Supplement: Additional file 2 — Similarity of the product of MsSN1 gene to other snakin/GASA proteins from plants. [file 12870_2014_248_MOESM2_ESM.doc]

**Additional File 2. Similarity of the product of *MsSN1* gene to other snakin/GASA proteins from plants.**

| Protein (accession number) | Amino acid identity (%) |
| --- | --- |
| *Medicago sativa* (AFE82743) | --- |
| *Medicago truncatula* (XP_003589114) | 97.8 |
| *Cicer arietinum* (XP_004499015) | 69.8 |
| *Lotus japonicus* (LJ5G003340) | 59.8 |
| *Glicine max* (GM06G04740) | 46.7 |
| *Manihot esculenta* (ME03237G00480) | 51.2 |
| *Ricinus communis* (XP_002510126) | 55.6 |
| *Populus trichocarpa* (XP_002327192) | 61.2 |
| *Theobroma cacao* (C02G009830) | 33.8 |
| *Carica papaya* (CP00292G00070) | 42.8 |
| *Arabidopsis lyrata* (XP_002883868) | 31.5 |
| *Arabidopsis thaliana* (AEC06348) | 31.7 |
| *Solanum tuberosum* (Q948Z4) | 43.2 |
| *Oryza sativa* IG ( AB192574) | 32.3 |
| *Oryza sativa* JG (NP_001051348) | 31.7 |
| *Zea mays* (NP_001149636) | 31.9 |
| *Setaria italica* (XP_004981680) | 27.5 |
| *Sorghum bicolor* (XP_002463849) | 30.4 |
| *Picea sitchensis* (ABK22434) | 31.1 |
| *Selaginella moellendorffii* (XP_002973082) | 37.0 |
| *Solanum tuberosum* (CAC44011) | 20.3 |

|
