# Supplementary material for: Alfalfa snakin-1 prevents fungal colonization and probably coevolved with rhizobia
Source: BMC Plant Biol. 2014 Sep 17;14:248. doi: 10.1186/s12870-014-0248-9 (PMC4177055; doi:10.1186/s12870-014-0248-9)
Supplement: Additional file 3 — Alfalfa transformation. [file 12870_2014_248_MOESM3_ESM.doc]

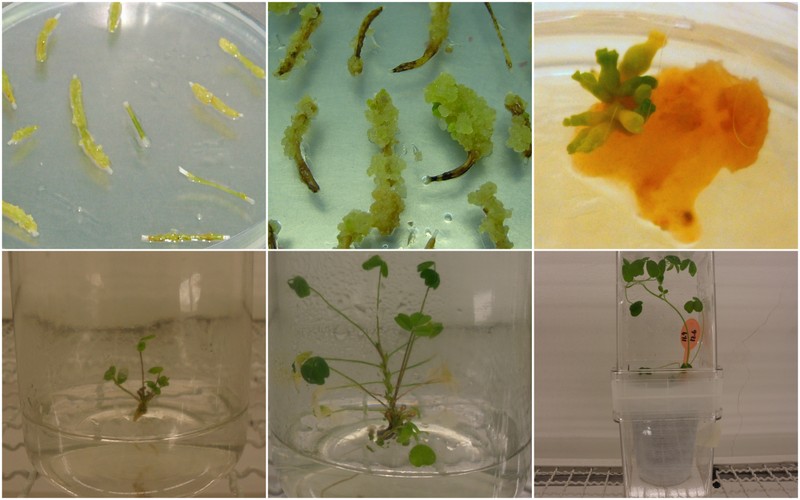


**A**

**B**

**C**

**D**

**E**

**F**

**Additional File 3: Alfalfa transformation protocol.** Representative pictures of alfalfa transformation assays. A. 15-day-old petioles in selective callus-inducing medium; B. 45-day-old petioles in selective callus-inducing medium; C. transgenic somatic embryos; D. transgenic regenerated plantlet growing in root-inducing medium; E. completed plantlet; F. three-month-old transgenic plant in magenta vessel.
