# Supplementary material for: Alfalfa snakin-1 prevents fungal colonization and probably coevolved with rhizobia
Source: BMC Plant Biol. 2014 Sep 17;14:248. doi: 10.1186/s12870-014-0248-9 (PMC4177055; doi:10.1186/s12870-014-0248-9)
Supplement: Additional file 4 — Identity of the product of MsSN1 gene to other snakin/GASA proteins from Arabidopsis. [file 12870_2014_248_MOESM4_ESM.doc]

**Additional File 4. Identity of the product of *MsSN1* gene to other snakin/GASA proteins from Arabidopsis.**

| Protein | Amino acid identity (%) |
| --- | --- |
| Ms (AFE82743) | ---- |
| GASA10 | 40.0 |
| GASA8 | 32.8 |
| GASA7 | 31.7 |
| GASA6 | 24.6 |
| GASA13 | 24.3 |
| GASA11 | 24.1 |
| GASA2 | 22.5 |
| GASA12 | 22.3 |
| GASA9 | 21.3 |
| GASA4 | 20.8 |
| GASA5 | 20.5 |
| GASA3 | 15.9 |
| GASA1 | 14.6 |
| GASA14 | 12.5 |


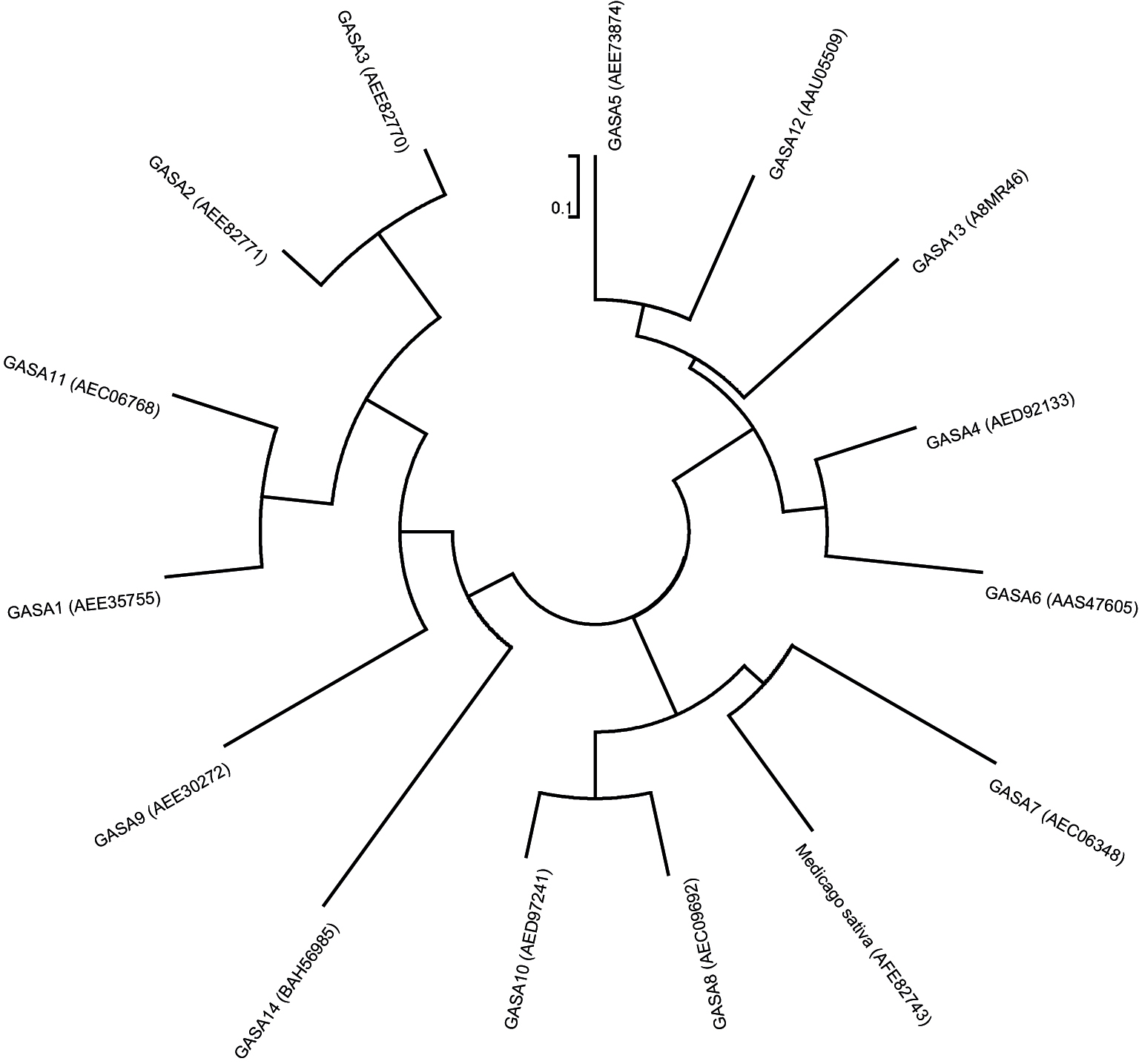


Phylogenetic tree of MsSN1 and GASA peptides from Arabdipsis using NJ-method and root on midpoint.
