# Supplementary figures and images for: Alfalfa snakin-1 prevents fungal colonization and probably coevolved with rhizobia
Source: BMC Plant Biol. 2014 Sep 17;14:248. doi: 10.1186/s12870-014-0248-9 (PMC4177055; doi:10.1186/s12870-014-0248-9)

**
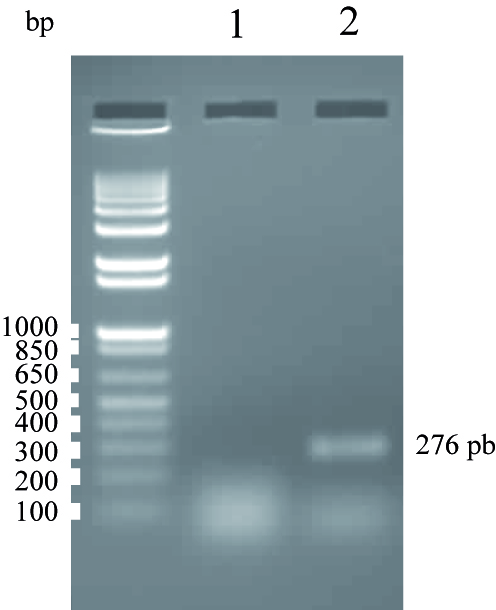
**

**Additional File 5.** RT-PCR studies of *MtSN1* gene expression in *E. coli* pSJ33 (1) and *E. coli* pSJ33-MsSN (2) strains.

Supplement: Additional file 5 — RT-PCR studies of MsSN1 gene expression in E. coli pSJ33 (1) and E. coli pSJ33-MsSN (2) strains. [file 12870_2014_248_MOESM5_ESM.doc]

**Additional File 8. Vigor phenotype of transgenic (S1) and wild-type (Wt) alfalfa plants.**


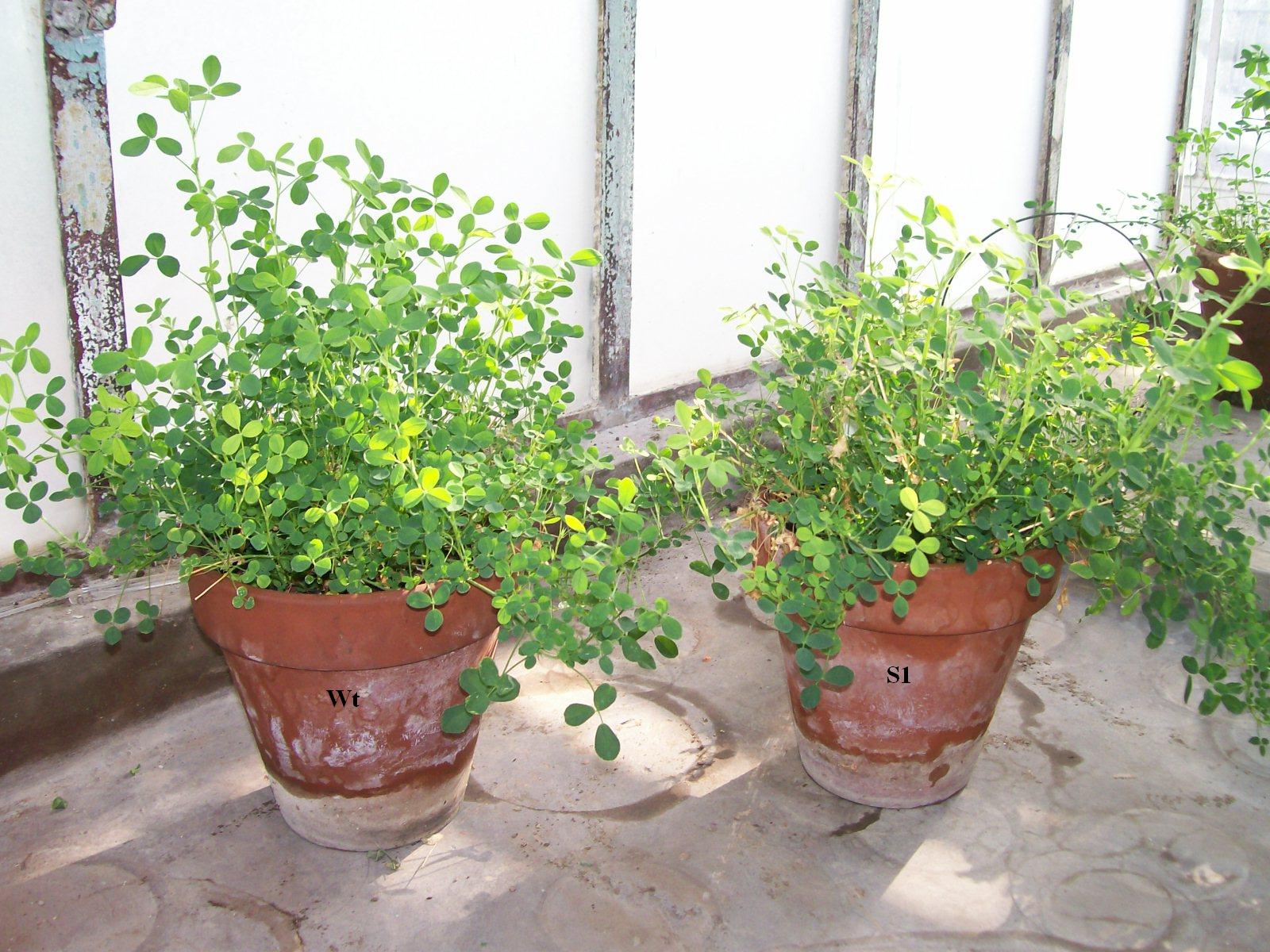

Supplement: Additional file 8 — Vigor phenotype of transgenic (S1) and wild type (Wt) alfalfa plants. [file 12870_2014_248_MOESM8_ESM.doc]
