# Supplementary material for: Alfalfa snakin-1 prevents fungal colonization and probably coevolved with rhizobia
Source: BMC Plant Biol. 2014 Sep 17;14:248. doi: 10.1186/s12870-014-0248-9 (PMC4177055; doi:10.1186/s12870-014-0248-9)
Supplement: Additional file 6 — RT-PCR assays showing MsSN1 expression in young floral buds (A-B), leaves (C-D), stems (E-F) and roots (G-H). [file 12870_2014_248_MOESM6_ESM.doc]

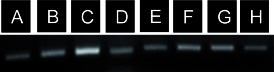


**276 bp**

**Additional File 6.** RT-PCR assays showing *MsSN1* expression in young floral buds (A-B), leaves (C-D), stems (E-F) and roots (G-H).
