# Supplementary material for: Alfalfa snakin-1 prevents fungal colonization and probably coevolved with rhizobia
Source: BMC Plant Biol. 2014 Sep 17;14:248. doi: 10.1186/s12870-014-0248-9 (PMC4177055; doi:10.1186/s12870-014-0248-9)
Supplement: Additional file 7 — Molecular characterization of transgenic alfalfa lines overexpressing MsSN1. [file 12870_2014_248_MOESM7_ESM.doc]

**Additional File 7.** Molecular characterization of transgenic alfalfa lines overexpressing *MsSN1* (S1-S3) and control untransformed plants (Wt). PCR assays (A); Southern Blot analyses using *MsSN1* cDNA labeled as probe (B); RT-PCR studies (C). Mix: negative controls using H_2_O instead of DNA (A) or RNA as templates (C). ATT: endogenous control gene. White arrows: Transgene insertion events in different regions of the alfalfa genome. 656 bp: CaMV35S:MsSN1 region; 429 bp: transgene; 763 bp and 386 bp: endogenous control gene ATT; Wt: wild-type alfalfa plants; Mix: negative controls using H_2_O, DNA (A) or RNA as templates ().

A

C

B

Transgene (5’topo-*MsSN1*)

CaMV35S:*MsSN1* region

*MsSN1* region

**
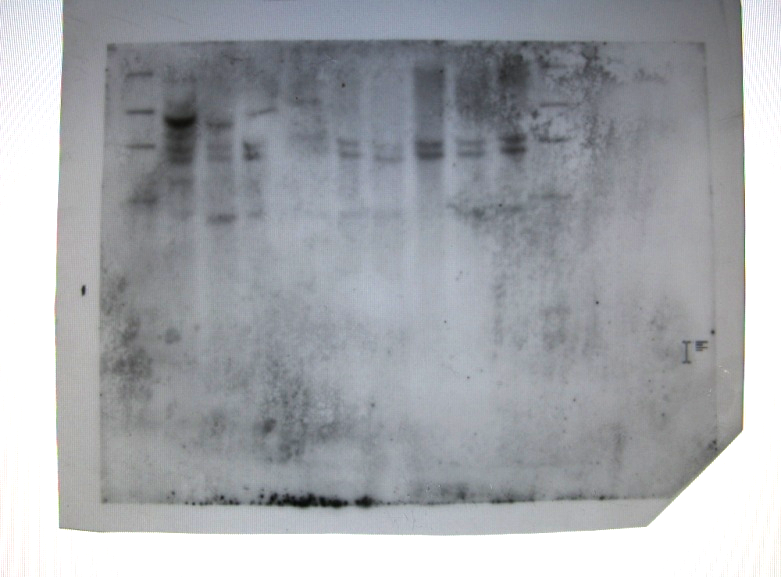

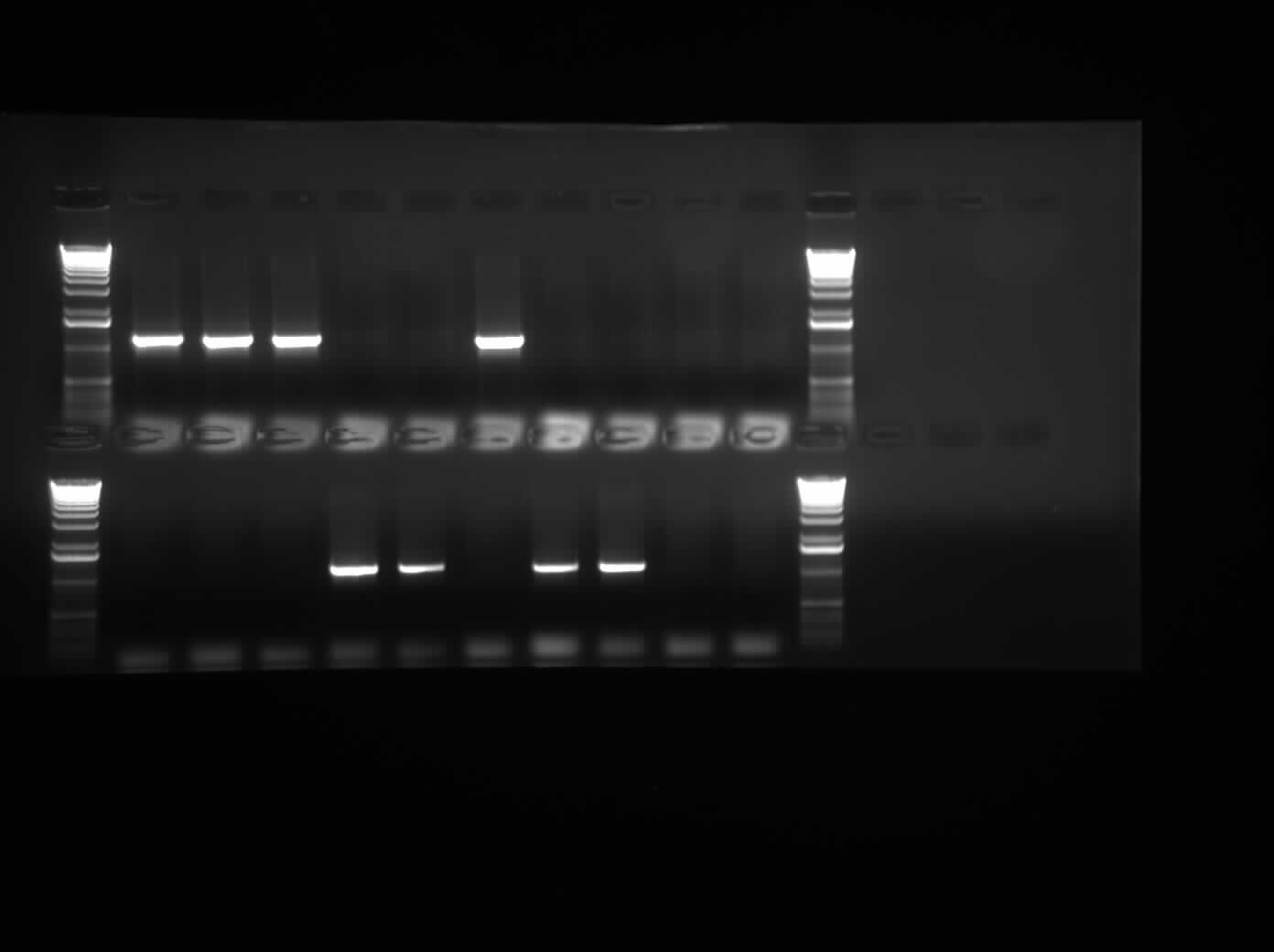

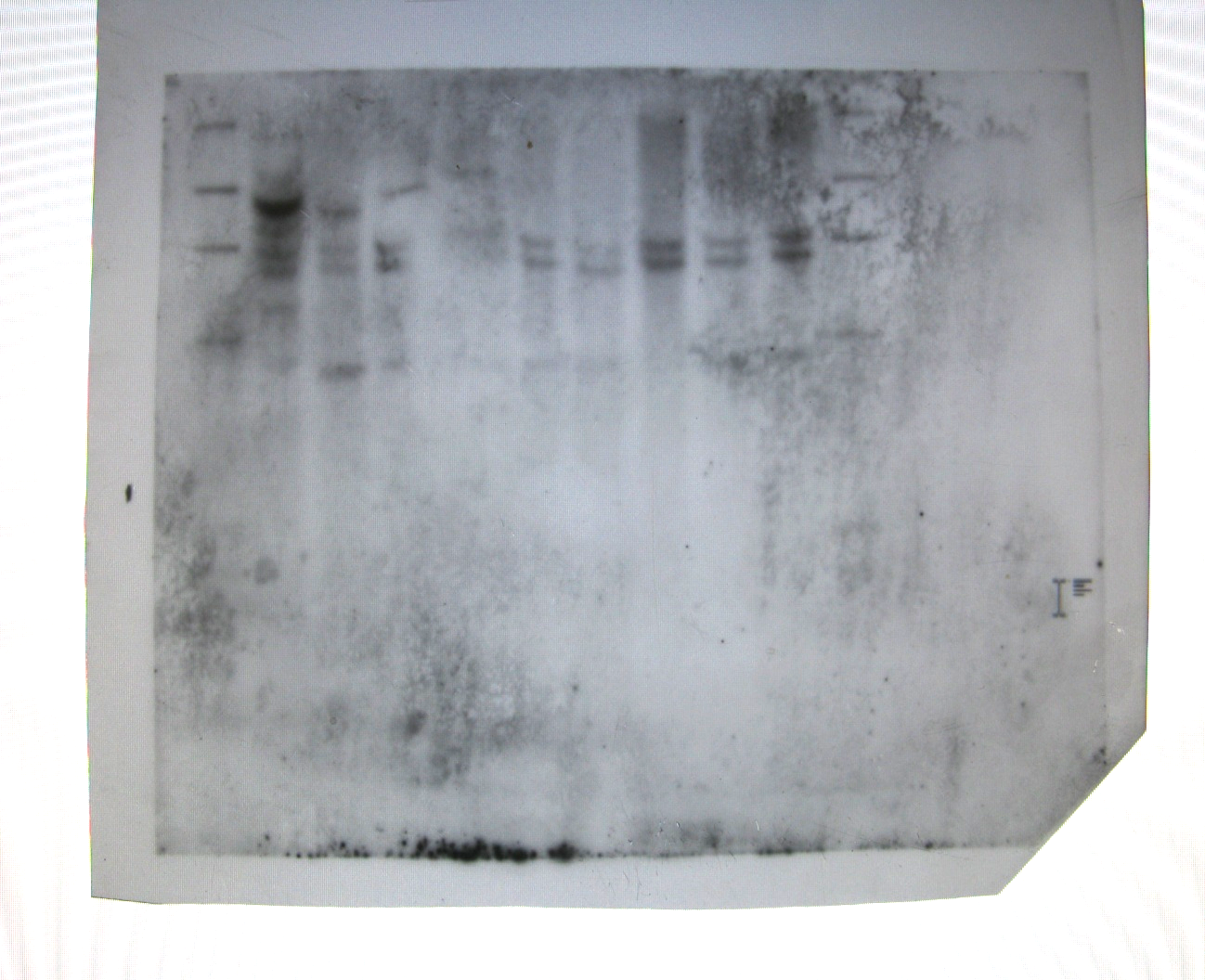

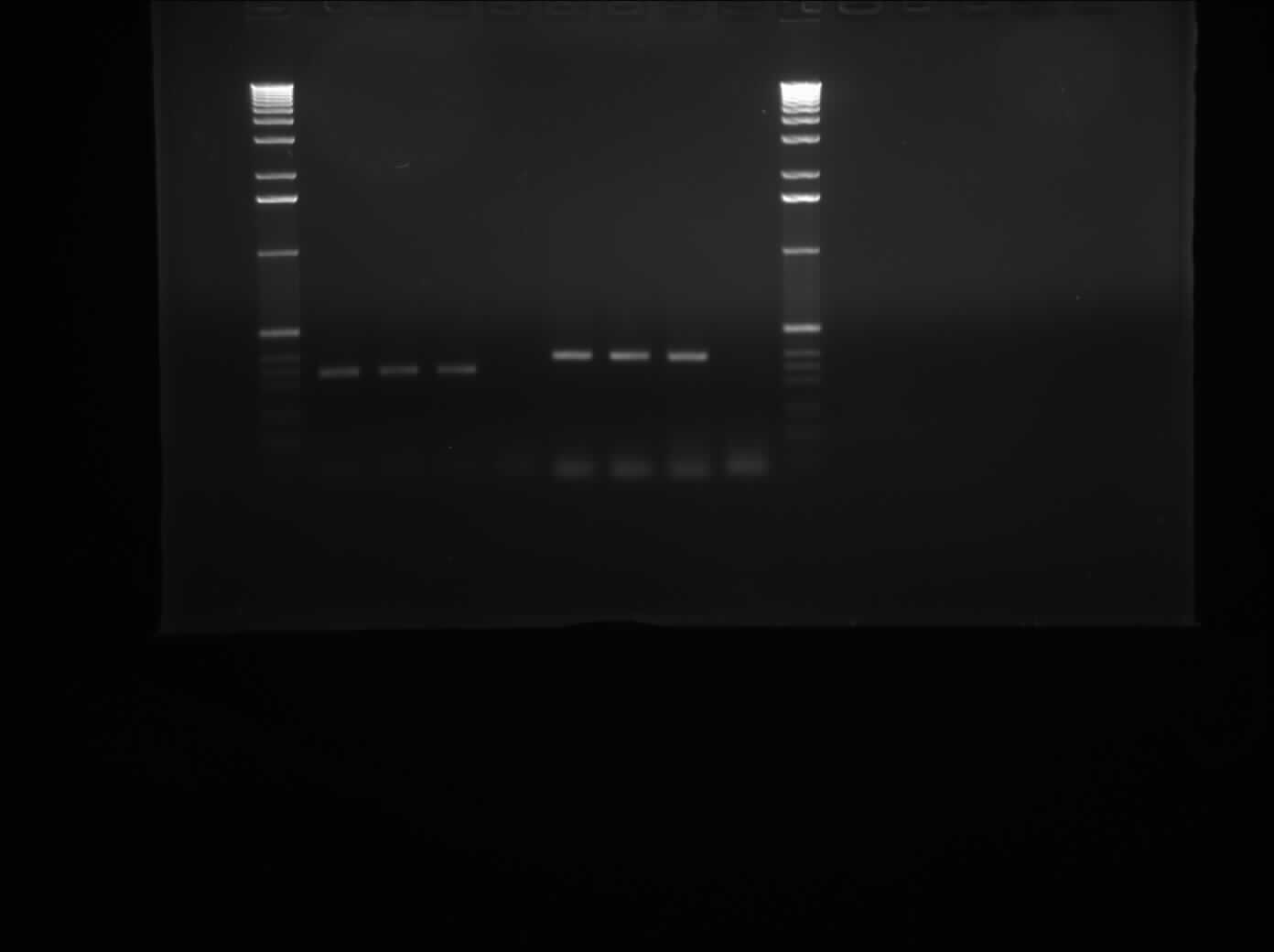

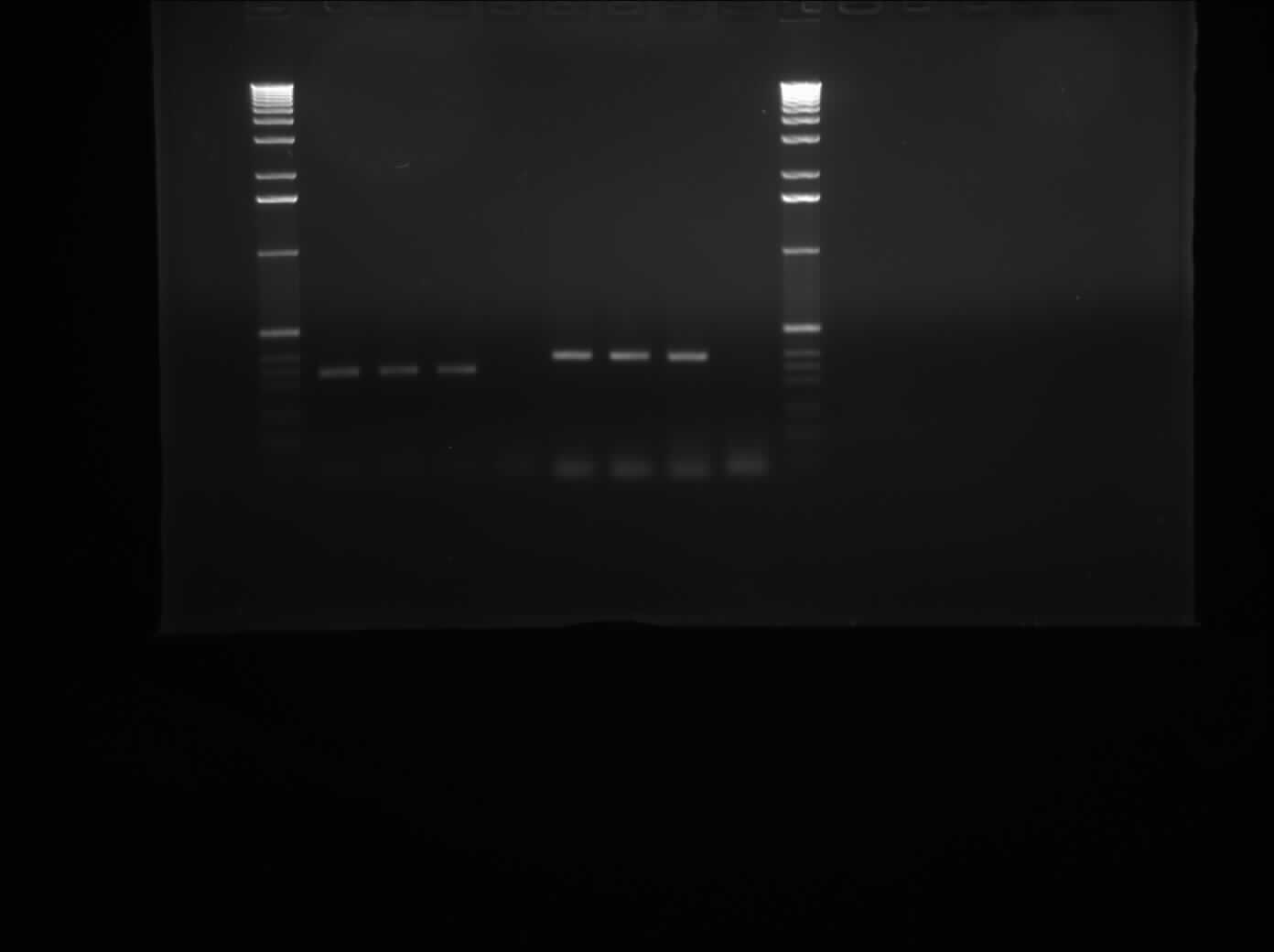
**

S1

S2

S3

656 bp

WtS1

mixx

S1

S2

S3

WtS1

mixx

21 kb

S1

S2

S3

WtS1

S1

S2

S3

mix

Wt

**
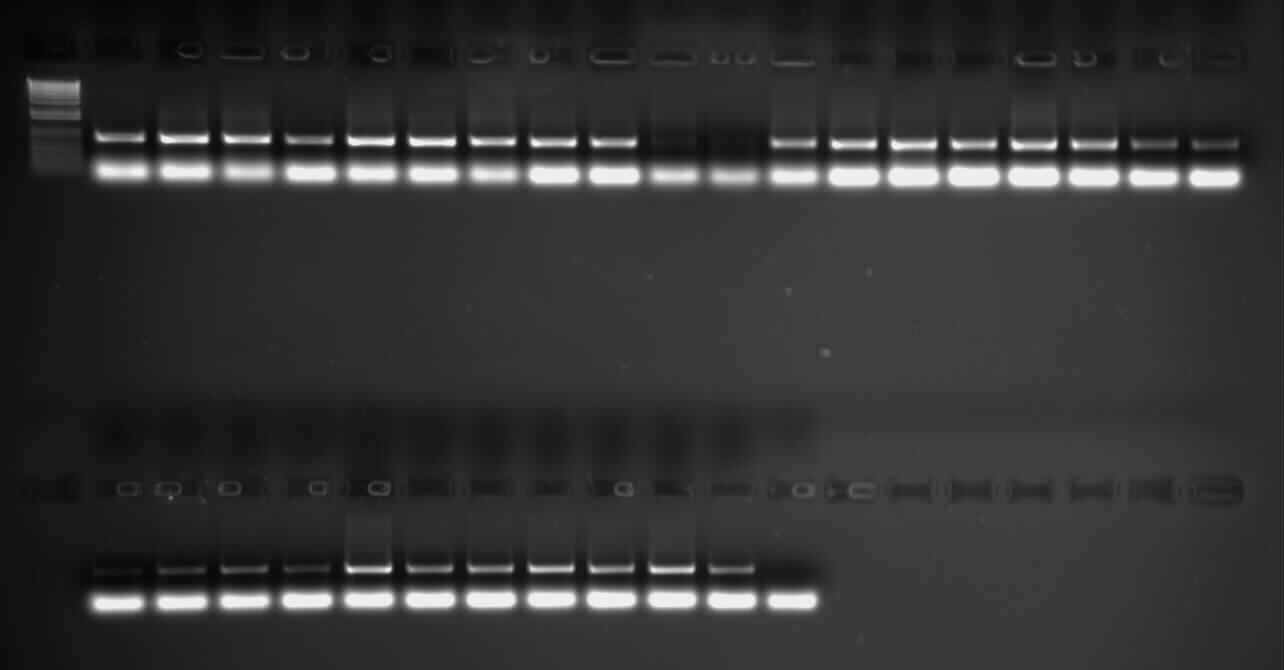

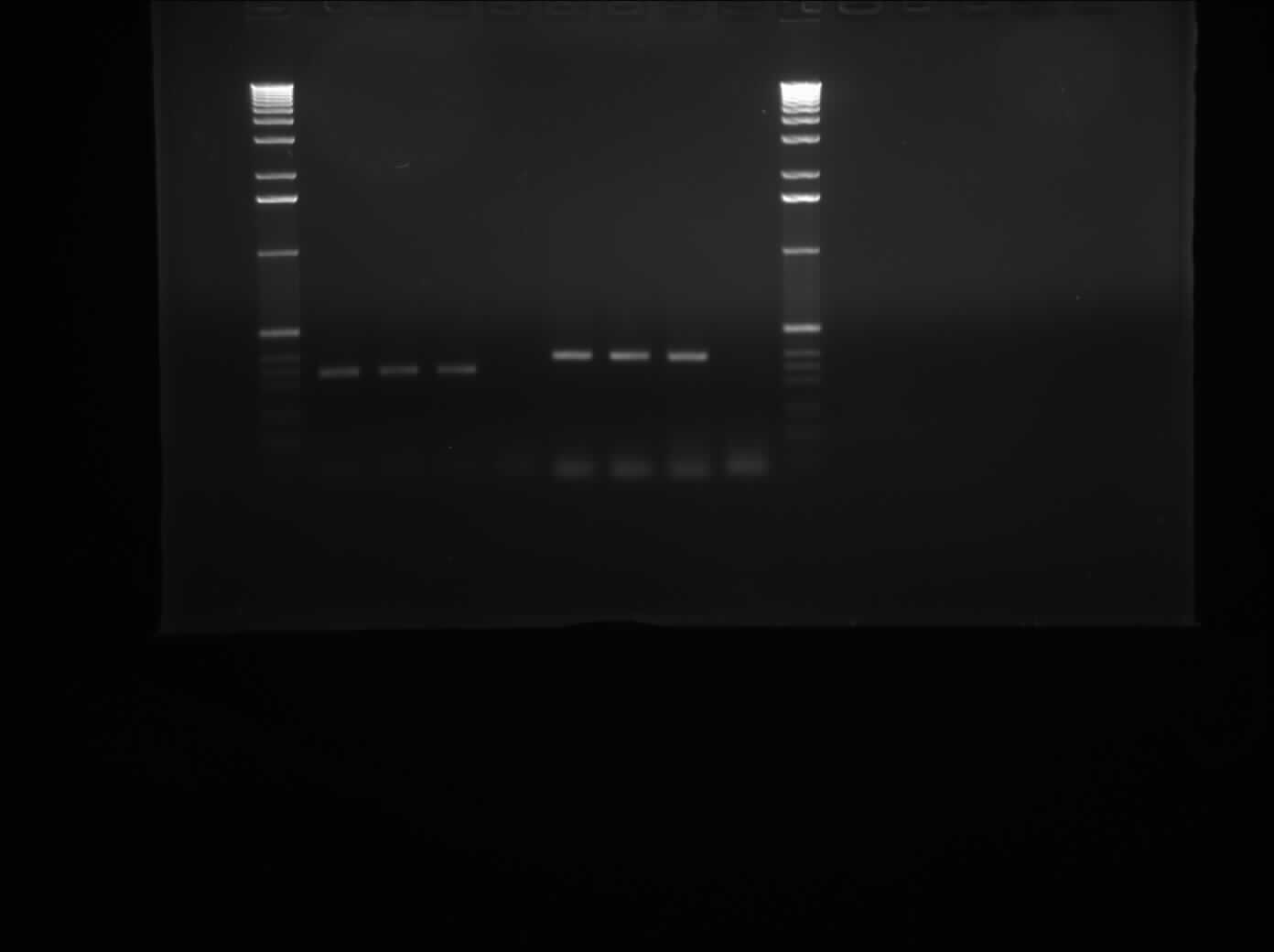

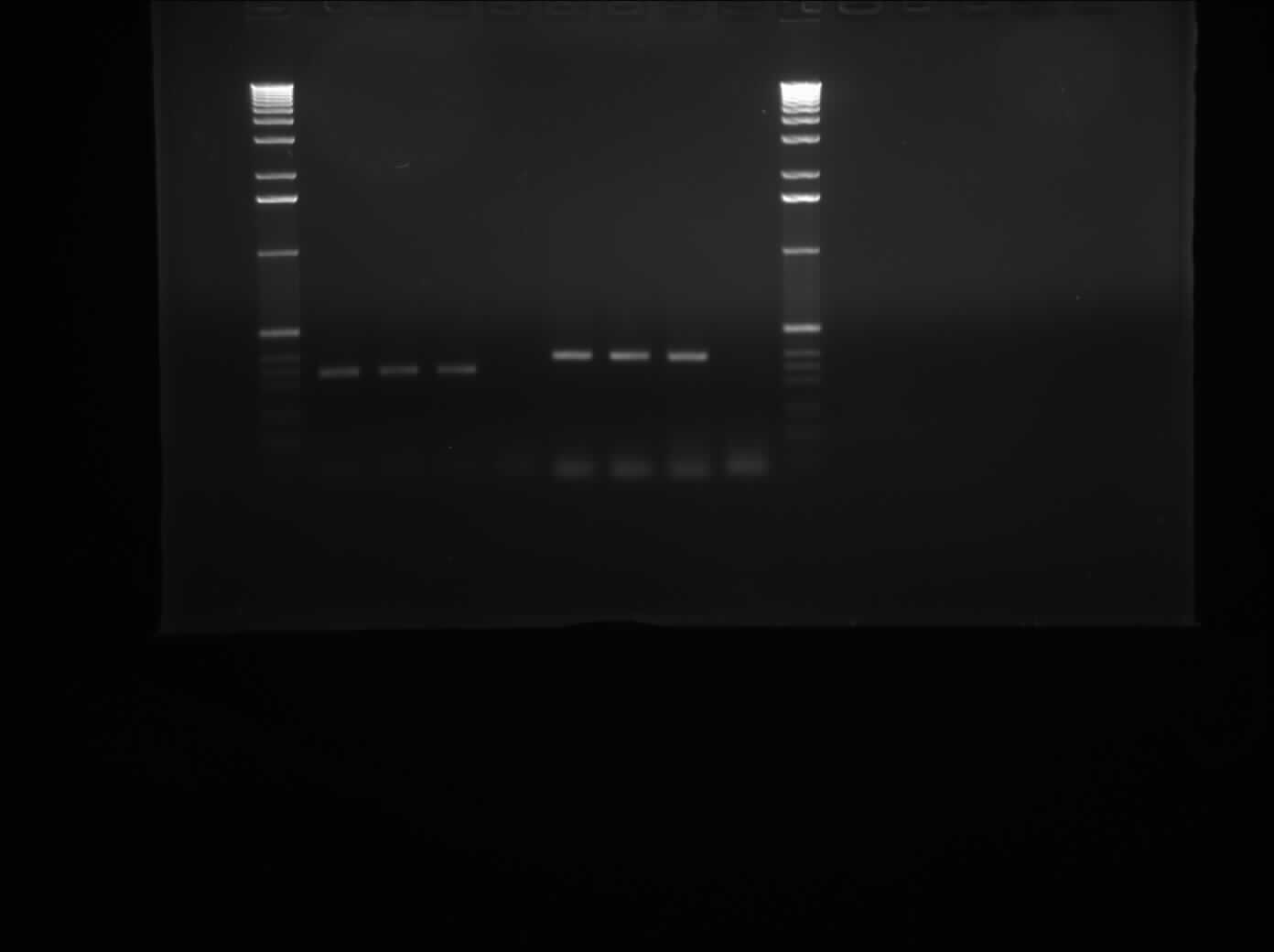
**

763 bp

ATT

5 kb

4.2 kb

3.5 kb

386 bp

ATT

429 bp
