# Supplementary material for: Alfalfa snakin-1 prevents fungal colonization and probably coevolved with rhizobia
Source: BMC Plant Biol. 2014 Sep 17;14:248. doi: 10.1186/s12870-014-0248-9 (PMC4177055; doi:10.1186/s12870-014-0248-9)
Supplement: Additional file 9 — Phylogenetic analysis of fungal strains isolated from alfalfa. [file 12870_2014_248_MOESM9_ESM.doc]

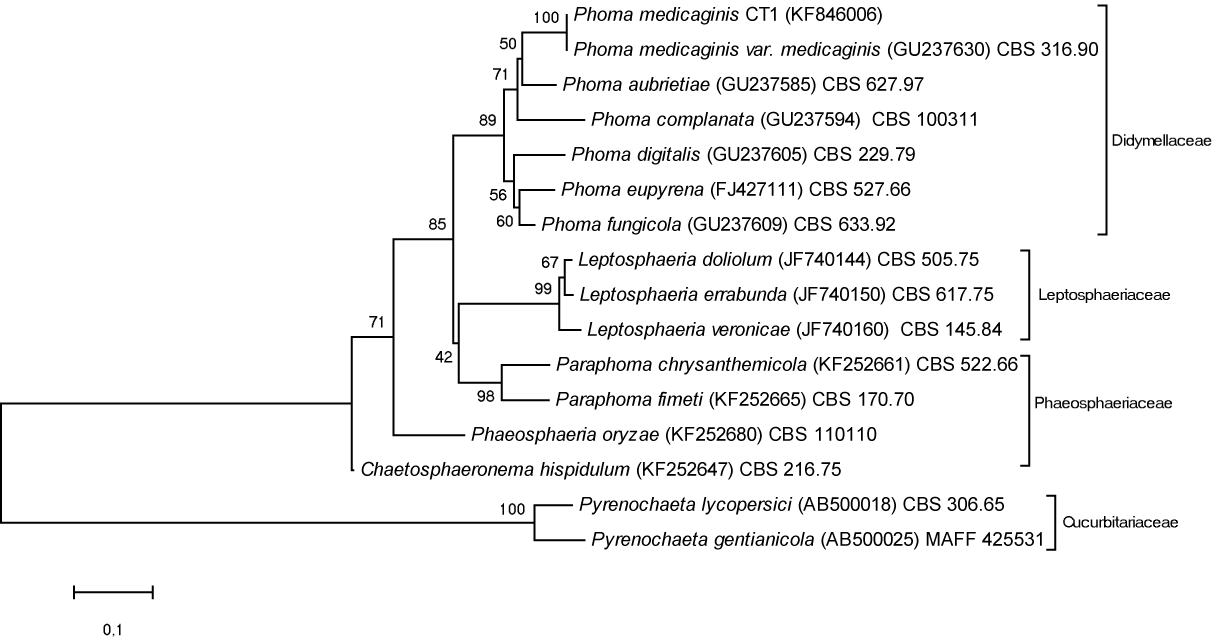


A


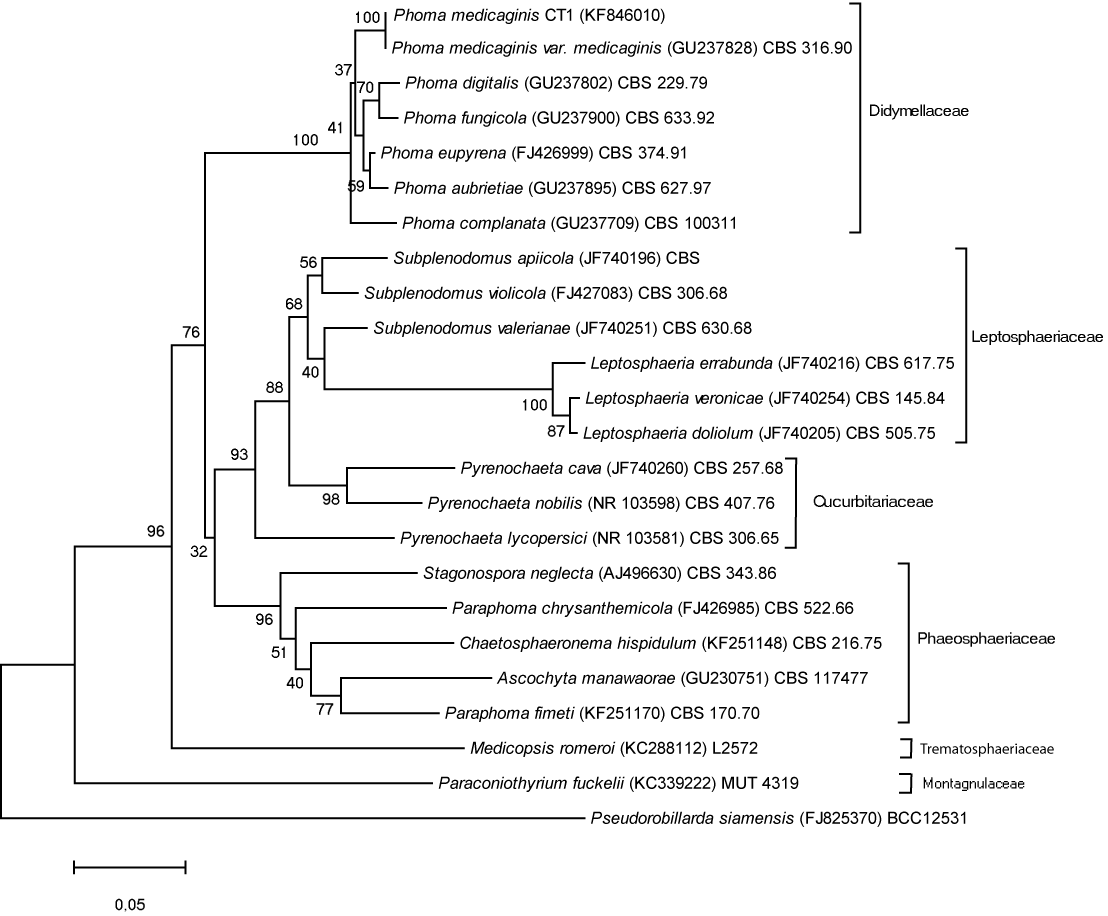

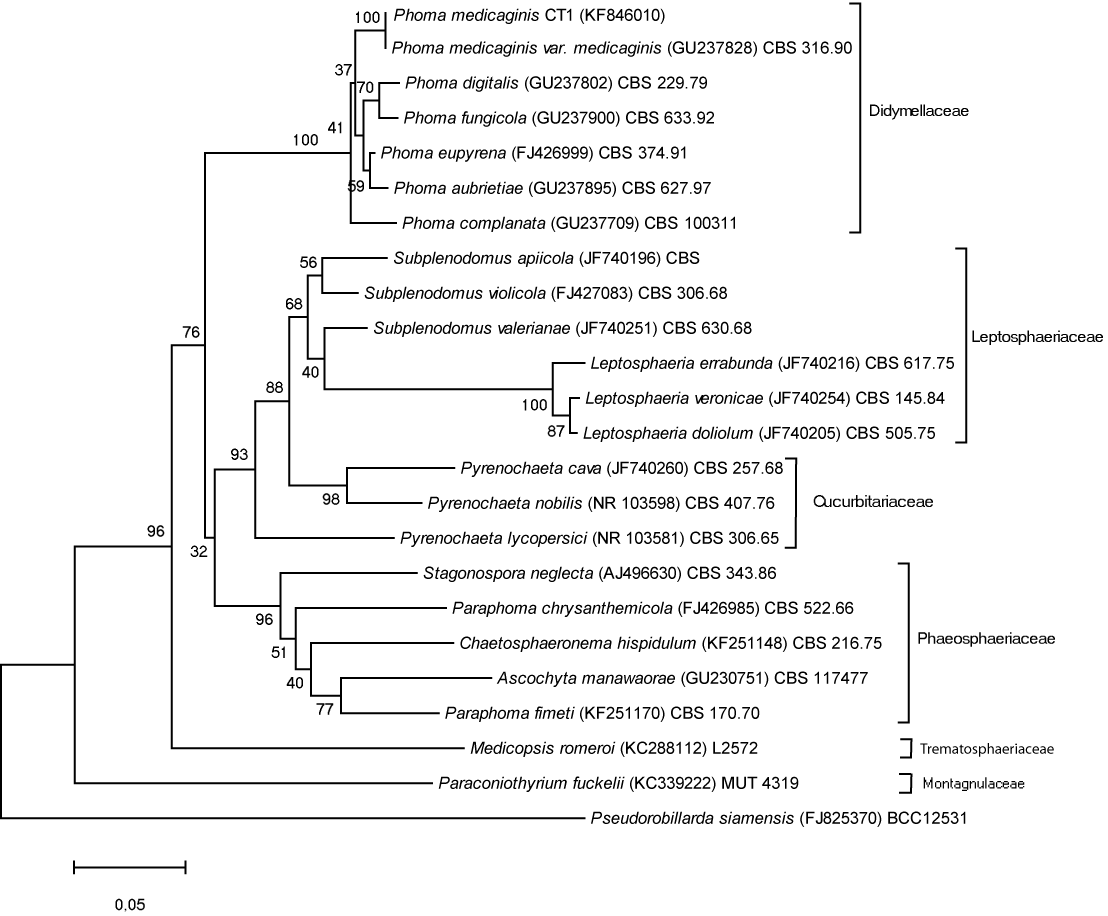


B

***Phoma medicaginis* CT1 (KF846006)**

***Phoma medicaginis* CT1 (KF846010)**

C

D


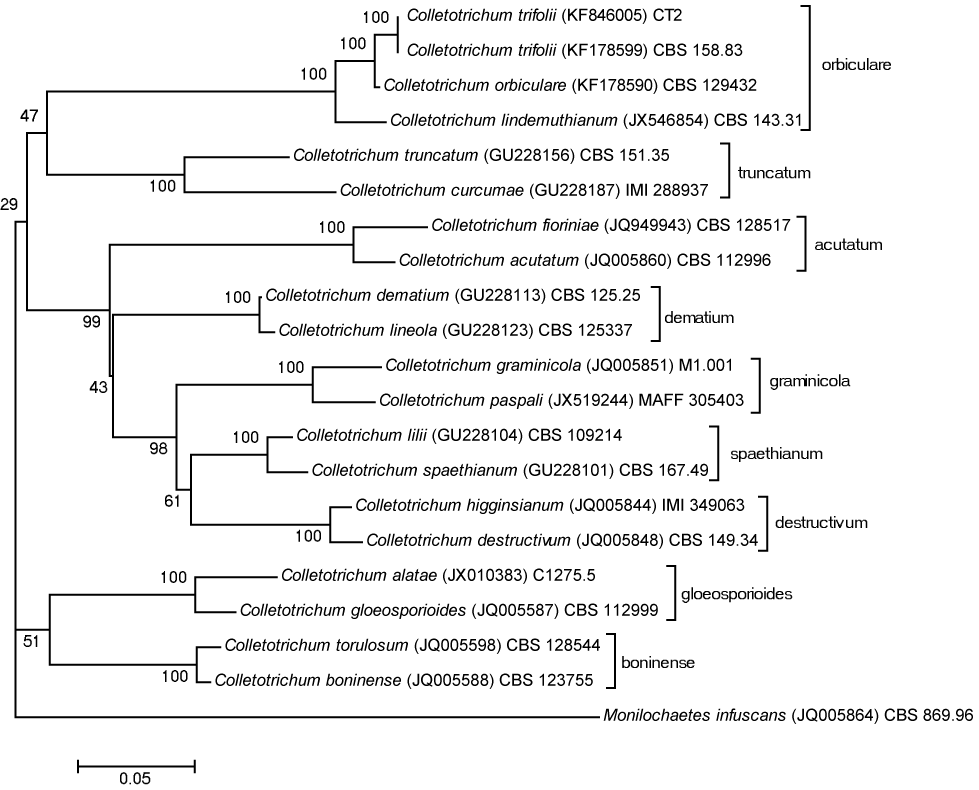

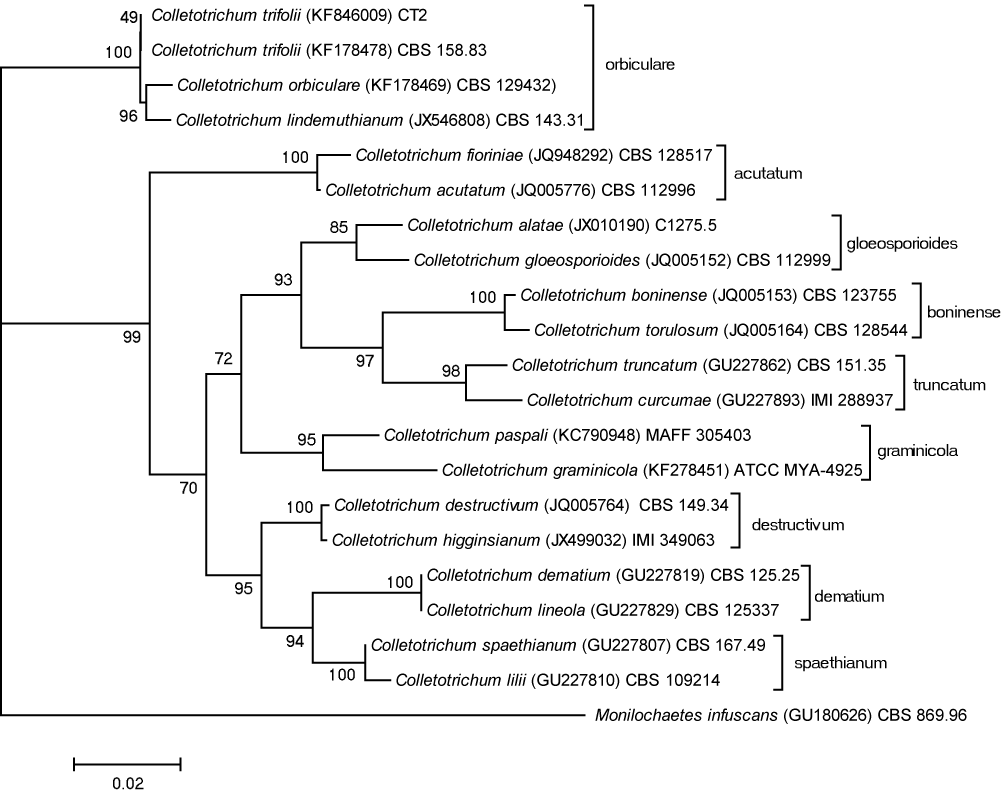


***Colletotrichum trifolii* CT2 (KF846009)**

***Colletotrichum trifolii* CT2 (KF846005)**

**Additional File 9. Phylogenetic analysis of fungal strains isolated from alfalfa.** Phylogenetic analysis of the ITS region (A) and TUB gene (B) from and the ITS region (C) and TUB gene (D) from *Colletotrichum trifolii* strain CT2 nucleotide sequences using the neighbor-joining method with genetic distances computed using the pairwise deletion model and bootstrap analysis of 500 samples and root on midpoint. Bootstrap percentages are indicated at the branch points. The current classification of fungal related-strains is found on the right.
